# Understanding Non-Covalent Interactions in Diphenyldiselenide and Diphenylselenide Cocrystals Using a Combined ^77^Se Magic-Angle Spinning Solid-State NMR and Quantum Chemical Analysis Approach

**DOI:** 10.1021/acs.jpcc.5c01979

**Published:** 2025-07-16

**Authors:** Alireza Nari, Sajesh P. Thomas, David L. Bryce, Brijith Thomas

**Affiliations:** † Department of Chemistry and Biomolecular Sciences, Centre for Catalysis Research and Innovation, and Nexus for Quantum Technologies, 6363 University of Ottawa, Ottawa, Ontario KIN6N5, Canada; ‡ Department of Chemistry, 28817 Indian Institute of Technology Delhi, New Delhi 110016, India; § Chemistry Program, Science Division and Mubadala Arabian Center for Climate and Environmental Sciences, 167632 New York University Abu Dhabi, Abu Dhabi 129188, United Arab Emirates

## Abstract

Chalcogen bonds are σ-hole interactions that arise from the net attractive forces between an electron-deficient chalcogen atom (such as selenium) and a Lewis base. In recent years, chalcogen bonds have become important noncovalent interactions, playing a key role in building supramolecular structures and functional materials. Given their significance, there is a continuous interest in gaining a deeper understanding of chalcogen interactions. In this study, we examined systems involving Se–I interactions, where diphenyldiselenide and diphenylselenide serve as selenium sources, while molecular iodine and 1,4-diiodotetrafluorobenzene act as iodine donors. We explore the intricate interplay between selenium’s chemical environment and its role in noncovalent interactions, with a focus on Se···I chalcogen bonds and halogen bonds. An interdisciplinary approach combining solid-state NMR, single-crystal X-ray diffraction, and advanced quantum chemical analyses, such as the Quantum Theory of Atoms in Molecules (QTAIM), Non-Covalent Interactions analysis (NCI), the Extended Transition-State Method with Natural Orbitals for Chemical Valence (ETS-NOCV), and Interactive Quantum Atoms (IQA), were used to investigate the electronic and structural factors influencing selenium’s behavior. By analyzing the chemical shift tensors, we demonstrate how they are influenced by both halogen and chalcogen bonding roles, in addition to the effects of crystal packing and weak interactions.

## Graphical abstract

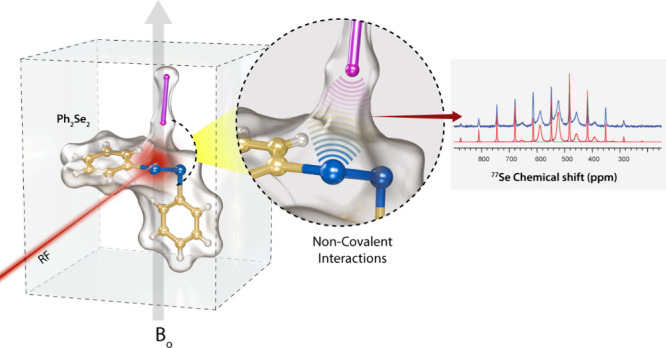

## Introduction

The adaptability of selenium, enabled by its wide range of accessible oxidation states from −2 to +6, makes it a versatile element in both covalent and noncovalent interactions.^1,2^ This chemical flexibility enables its crucial roles across a range of areas, including synthetic chemistry, medicinal chemistry, and materials science.^3–7^ Selenium is not only central to bioactive compounds but also emerges as a key player in noncovalent interactions, such as selenium-halogen (Se···X) (X: Cl, Br, I) interactions, which are integral to supramolecular and materials chemistry.^3–7^ These interactions exhibit unique versatility: in some cases, halogen atoms (X) function as σ-hole donors with selenium as the halogen bond (XB) acceptor, while in other instances, selenium itself can function as a σ-hole donor, forming chalcogen bonds (ChBs) with halogen acceptors.^8,9^ The broad chemical shift range of ^77^Se NMR provides a powerful tool for discerning subtle variations in bonding and geometry, enabling precise characterization of selenium-containing cocrystals. By examining chemical shift tensors, it is possible to reveal the dependence on halogen and chalcogen bonding roles, as well as the influence of crystal packing and weak interactions. The interaction between selenium and N-heterocyclic carbene ligands provides valuable insights into the electronic properties of these ligands, often probed through selenium solution state NMR chemical shifts.^10–12^

A XB is a directional, noncovalent interaction involving an attractive force between a halogen atom and a nucleophile. This interaction is driven by electrostatic attraction between the nucleophile and a region of elevated electrostatic potential (σ-hole) on the halogen, oftentimes alongside a charge-transfer from the nucleophilic lone pair to the antibonding σ* orbital of the halogen.^7–11^ Halogen bonding closely parallels hydrogen bonding in many respects. The σ-hole in halogen bonds functions similarly to the proton in hydrogen bonds (HBs), enabling precise and tunable noncovalent interactions.^12,13^

Well-known XB acceptors include nitrogen, oxygen, sulfur, and halogens themselves,^14–21^ along with some less common acceptors like phosphorus^22^ and halide anions. Selenium, however, is among the less-studied XB acceptors.^23^ Belonging to Group 16, known as the chalcogens, selenium can exhibit amphiphilic Lewis character, playing a dual role in the noncovalent interaction world as both a Lewis acid and a Lewis base when interacting with halogen atoms.^24,25^ In ChB, the σ-hole is positioned along the extension of the R–Se covalent bond.^26^ This property can be a powerful tool in the design of (supra)­molecular materials, bearing specific properties similar to other well-known σ-hole interactions like HB and XB. The presence of electron withdrawing groups (EWGs) on potential σ-hole donors plays a crucial role in defining the interaction characteristics, determining which component acts as the Lewis acid and which as the Lewis base, considering the amphiphilic nature of both halogen and chalcogen atoms.^27,28^

Selenium–halogen (Se···X) noncovalent interactions cover a broad range of applications in supramolecular design, biochemistry, catalysis, and optoelectronics, where selenium can act as either a σ-hole donor or acceptor.^24,29^ Certain chalcogen-containing compounds, such as 1,2,5-chalcogenadiazoles (Ch = S, Se, Te), are especially recognized as robust and versatile Lewis bases; substituent variations allow fine-tuning of σ-hole strength and, consequently, the strength of ChB interactions.^30–34^ Gaining a deeper understanding of the Se–I interaction is therefore crucial for advancing knowledge across various domains. Herein, we investigate the Se–I interaction in a series of molecular systems, as depicted in Scheme 1. Specifically, diphenyldiselenide (**1**) and diphenylselenide (**2**) serve as selenium sources, while iodine (**a**) and 1,4-diiodotetrafluorobenzene (*p*-DITFB) (**b**) act as iodine sources. Through a combination of experimental solid-state NMR (SSNMR), single-crystal X-ray diffraction (SCXRD), and advanced quantum chemical analyses such as the Quantum Theory of Atoms in Molecules (QTAIM), noncovalent interaction (NCI) analyses, and Extended Transition State–Natural Orbitals for Chemical Valence (ETS-NOCV) analyses, we investigate the electronic and structural factors influencing selenium’s behavior and its role in Se···I chalcogen bonding and halogen bonding. This work highlights the dual role of selenium as a ChB donor and XB acceptor, as well as shedding light on Se–Se and Se–Ph interactions, enhancing our understanding of selenium’s reactivity and its significance in noncovalent chemistry.

**Scheme 1:**
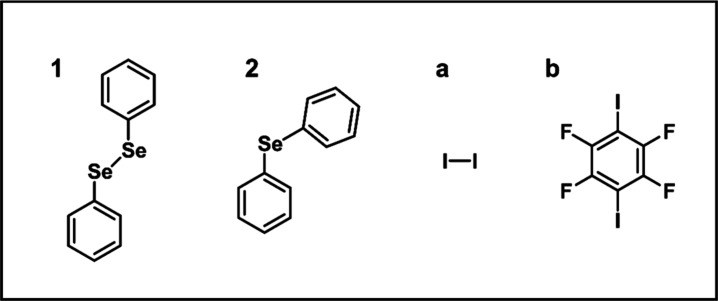
Molecular Structures of the Donors and Acceptors Used in This Study

## Results and Discussion

### X-ray Diffraction and Structural Features

Cocrystals **1a**, **1b**, and **2b** were synthesized via mechanochemical methods and subsequently characterized to confirm their phase purity and crystallographic integrity. Specifically, **1a** is a cocrystal formed from diphenyldiselenide (**1**) and iodine (**a**) in a **1:1** ratio, **1b** is a cocrystal of diphenyldiselenide (**1**) and *p*-DITFB (**b**) in a **1:2** ratio, and **2b** consists of diphenylselenide (**2**) and *p*-DITFB (**b**) in a **1:1.5** ratio.

As shown in Figure 1, the experimental powder X-ray diffraction (PXRD) patterns of the bulk samples closely match the simulated patterns derived from SCXRD data, confirming that the synthesized samples retained the expected crystallographic structure. This detailed comparison ensures the absence of significant impurities or secondary phases and verifies that the observed structural and interaction data, including those involving selenium and iodine, are intrinsic to the intended crystalline form.

**Figure 1:**
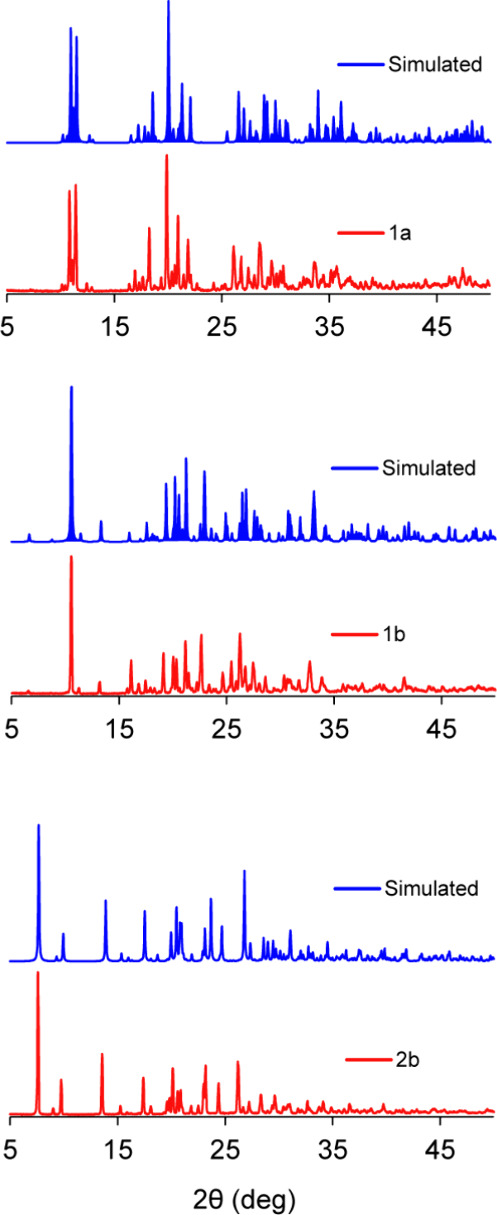
Comparison between experimental PXRD patterns of cocrystals prepared in this work (red) and their corresponding simulated diffractograms (blue).

In the cocrystal of diphenyldiselenide and molecular iodine (**1a**), a previously reported structure,^35^ distinct Se···I interactions illustrate the versatile donor–acceptor roles of selenium and iodine. In this structure, one selenium atom (Se1 or Se5, Figure 2a) acts as an electron donor to an iodine atom within the diiodine molecule, engaging in a XB interaction. This interaction involves selenium providing electron density toward the σ-hole of iodine, stabilizing the noncovalent bonding. Simultaneously, the other selenium atom in **1** plays an acceptor role by interacting with an iodine atom of a neighboring diphenyldiselenide moiety, here participating in a ChB. In this second interaction, selenium accepts electron density from the electron-rich region of the iodine atom, facilitated by the linear I···Se—Se and Se···I—I arrangements characteristic of hypervalent bonding, as discussed further in the QTAIM analysis section (vide infra). These dual donor–acceptor interactions exemplify selenium’s amphiphilic nature and underscore the importance of selenium–iodine noncovalent bonding in supramolecular assembly.

**Figure 2:**
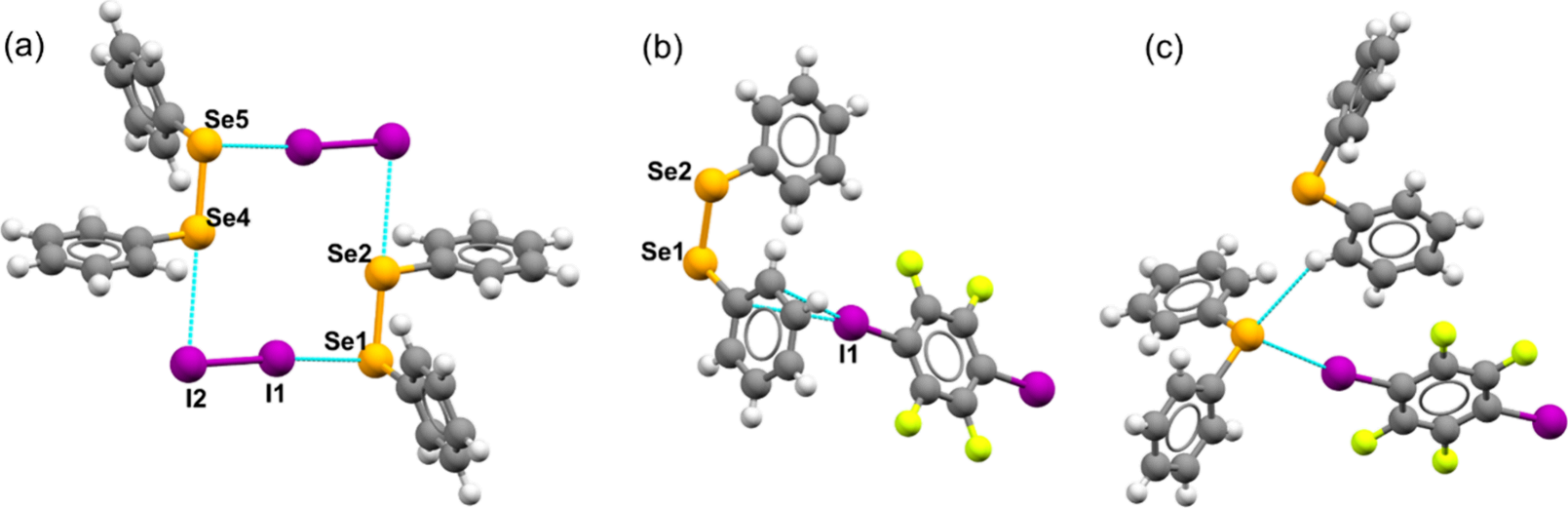
Crystal structures of (a) **1a**, (b) **1b**, and (c) **2b**, with selected atoms labeled, highlighting the diversity of bonding environments observed for selenium.

In cocrystal **1b**, two selenium atoms are present: one (Se1, Figure 2b) is bonded to the phenyl ring involved in a XB with *p*-DITFB, while the other Se, (Se2) is attached to a phenyl ring with no halogen bond interaction. The electronic environment of these two Se atoms, influenced by their respective phenyl rings, warrants further investigation to elucidate their roles. This analysis is crucial for determining whether each Se atom acts as a ChB σ-hole donor or a ChB acceptor in interactions with adjacent diphenyldiselenide molecules. This will be further analyzed in the upcoming sections.

Table 2 summarizes critical bond distances, interaction angles, and interaction types for compounds **1**, **1a**, **1b**, and **2b**, illustrating the influence of various cocrystallized molecules on the Se–Se covalent bond in **1**, diphenyldiselenide. In **1**, the Se–Se bond distance is 2.307 Å, reflecting a typical value for a diselenide. Upon cocrystallization with iodine (cocrystal **1a**), this distance increases to 2.347 Å. This elongation can be rationalized by considering orbital interactions and charge-transfer effects: the selenium lone pair orbitals donate electron density into the electrophilic region of the iodine moiety, likely involving the σ* orbitals of the I–I bond or other vacant orbitals on iodine. This electron donation depletes electron density from the Se–Se bond, weakening and lengthening it. The ETS-NOCV analysis quantifies these interactions, revealing significant orbital contributions in **1a**: Δ*E*_σ1_= −23.99 kcal/mol, Δ*E*_σ2_ = −3.85 kcal/mol, and Δ*E*_π1_ = −1.75 kcal/mol (see Figure S1­(i) in the Electronic Supporting Information, ESI). The dominant Δ*E*_σ1_ term reflects a strong σ-type charge-transfer interaction, consistent with the observed XB and ChB (at 2.99 and 3.59 Å), which redistributes electron density away from the Se–Se bond. This is shown in Figure S1­(i), where the NOCV orbital pairs show significant electron depletion (blue lobes) around the Se–Se bond and accumulation (red lobes) toward the iodine acceptor, directly corroborating the bond elongation from 2.307 to 2.347 Å. In **1a**, the partial redistribution of electron density associated with these interactions reduces the electron density localized in the Se–Se bond, subtly weakening and elongating it, as reflected by its length of 2.35 Å in Table 2, which is longer than in **1** and **1b**.

In contrast, for the **1b** cocrystal with *p*-DITFB, no direct Se···I short contacts are observed. Instead, the halogen bond interacts with the π-system of the phenyl ring. Although electron redistribution still occurs, the electrophilic demand from the π-system is lower than that from I_2_, resulting in a minimal increase in the Se–Se bond distance to 2.313 Å, only slightly longer than in pure **1**. The ETS-NOCV analysis for **1b** supports this weaker interaction, with Δ*E*_σ1_ = −1.25 kcal/mol, Δ*E*_σ2_ = −0.46 kcal/mol, and Δ*E*_π1_ = −0.72 kcal/mol (see Figure S1­(ii)). These smaller Δ*E* values indicate less significant charge transfer compared to **1a**, consistent with the reduced impact on the Se–Se bond. Figure S1­(ii) further illustrates this, showing less pronounced electron depletion around the Se–Se bond compared to **1a**, aligning with the weaker influence of the π-system interaction.

The crystal structure of cocrystal **2b,** reported here for the first time (Figure 2c) was solved in the monoclinic space group *P*2_1_/*n* with cell dimensions *a* = 13.011 Å, *b* = 6.148 Å, *c* = 18.166 Å and a cell volume of 1422.46 Å^3^ (Table 1). The structure reveals notable selenium–iodine (Se···I) and selenium–hydrogen (Se···H) interactions. The Se···I interaction, with a distance of 3.551 Å, falls within the range typical of XB, where iodine acts as a σ-hole donor and selenium serves as a XB acceptor. Additionally, the selenium atom forms a secondary interaction with a hydrogen atom from a neighboring diphenylselenide molecule, with a Se···H distance of 3.053 Å, indicating a weak hydrogen bond. These dual interactions — Se···I as a halogen bond and Se···H as a weak hydrogen bond (Figure 2c)— highlight selenium’s amphiphilic nature, allowing it to participate in both chalcogen bonds and hydrogen bonding-like interactions (vide infra), which collectively enhance the stability of the cocrystal structure.

**Table 1:** Crystallographic Data for **2b**

compound	**2b**
chemical formula	C_30_H_20_F_4_I_2_Se_2_
CCDC number	2416743
*FW*/g mol	868.18
crystal color	clear
crystal system	monoclinic
crystal space group	*P*2_1_/*n*
temperature/K	100
*a*, *b*, *c*/A°	13.0111(5), 6.1482(2), 18.1659(6)
α, β, γ/°	90, 101.802(2), 90
*V*/A°^3^	1422.46 (9)
*Z*	2
*R*_1_ (final)	0.0258
*wR*_2_ (final)	0.0534

**Table 2:** Selected Non-Covalent Interaction Distances to Se, Se–Se Covalent Bond Lengths (in Å), and Interaction Angles (in Degrees) for Compounds, **1**, **1a**, **1b**, and **2b**

compound/CSD refcode	Se–Se covalent bond length	noncovalent interaction distance/interacting atom	noncovalent interaction angle
**1**/DPHDSE02 ^36^	2.307(1)	3.778/Se	122.66[C–Se···Se]
		3.664/Se	172.35[C–Se···Se]
**1a /** GIHZIG^37^	2.347(2)	2.992/I	174.21[I–I···Se]
		3.588/I	176.50 [Se–Se···I]
**1b**/POGZOD^38^	2.313(1)	3.606/Se	94.76[C–Se···Se]
		3.752/Se	171.85[C–Se···Se]
**2b**/This work^a^		3.551/I	167.86[C–I···Se]

a CSD deposition number 2416743.

### Solid-State NMR Spectroscopy

Selenium-77 (^77^Se) stands out among selenium isotopes as NMR-active (*I* = 1/2) and naturally abundant at 7.58%. Notably, its NMR receptivity relative to ^13^C is approximately 2.9, making ^77^Se nearly three times more sensitive than ^13^C. This heightened sensitivity, combined with its broad chemical shift range of over 3000 ppm,^39,40^ renders ^77^Se NMR an invaluable tool for probing noncovalent interactions involving selenium. Such an extensive range allows for precise detection of subtle variations in oxidation states, coordination geometries, and ChB environments. Consequently, ^77^Se SSNMR is exceptionally well-suited for distinguishing nuanced electronic differences in organoselenium compounds. The sensitivity of ^77^Se chemical shifts to variations in local geometry and intermolecular interactions makes it challenging to assign ^77^Se solid-state NMR spectra based solely on chemical or molecular structure. To address this complexity, computational methods for calculating ^77^Se NMR parameters offer valuable insights for interpreting experimental data. In this study, density functional theory (DFT) calculations of ^77^Se magnetic shielding tensors were performed for all the compounds. A comparison of experimental and calculated selenium chemical shifts is presented in Table 4. Due to the presence of different short contacts in each molecule and the high sensitivity of ^77^Se chemical shifts to the local environment, a degree of scatter is observed between experimental and calculated values. Notably, a significant shift in the δ_22_ component of the chemical shift tensor is attributed to the short Se···I distance, with this contribution diminishing as the Se···I distance decreases. The ^1^H → ^77^Se cross-polarization magic-angle spinning NMR spectra for **1**, **1a**, **1b**, and **2b** are shown in Figure 3 (see also Figures S2 and S3). Diphenylselenide (**2**) was not included in Figure 3 as it is a liquid. The ^77^Se chemical shifts were referenced to solid (NH_4_)_2_SeO_4_ at δ_iso_ = 1040.2 ppm and are listed in Table 3.

**Figure 3:**
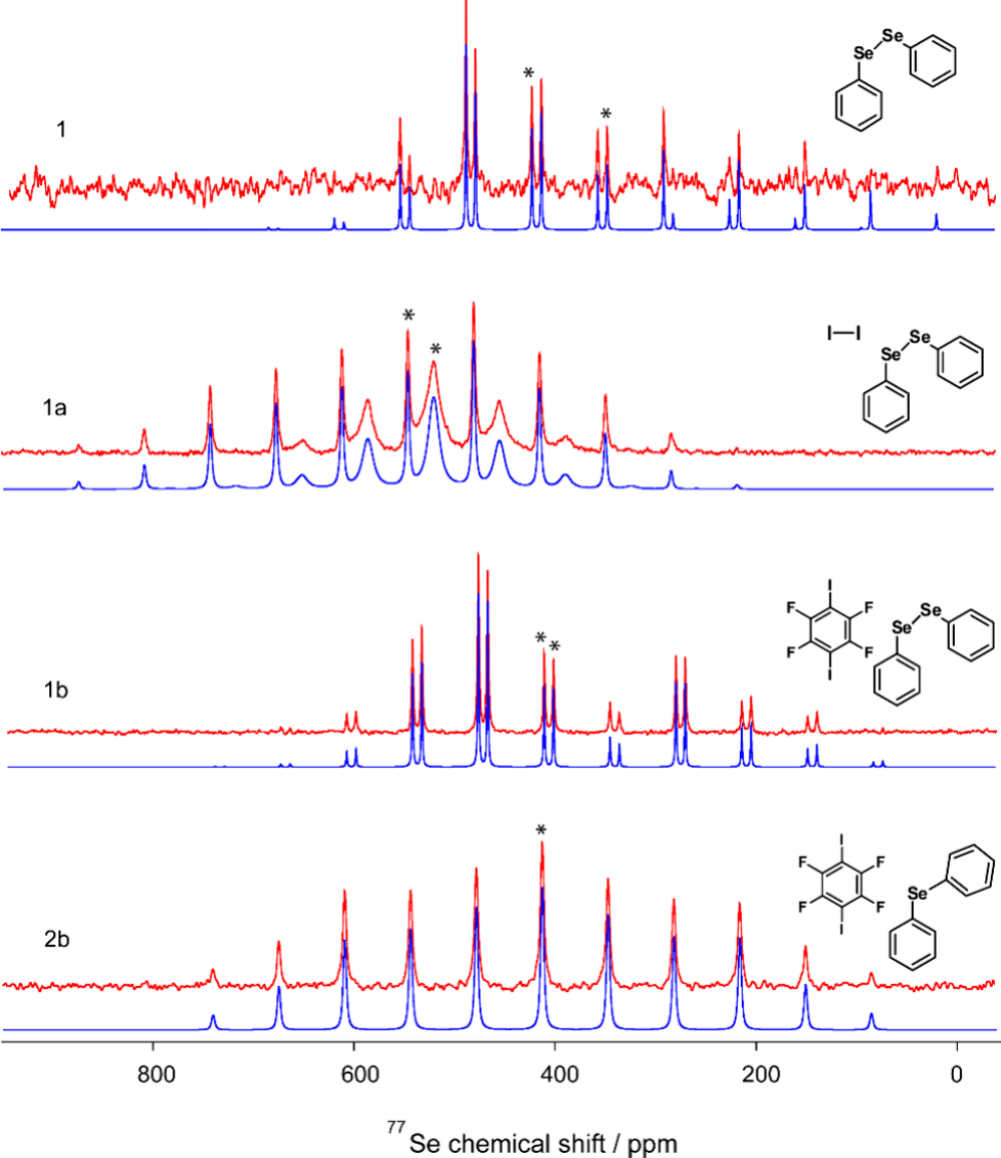
Experimental ^1^H → ^77^Se CP/MAS NMR spectra of diphenyldiselenide (**1**) and the studied cocrystals, collected at a spinning speed of 5 kHz (top, red), overlaid on their corresponding SOLA-simulated spectra (bottom, blue). The molecular structures of the selenium-containing species and each iodinated counterpart are shown above their respective spectra. Isotropic peaks are indicated with asterisks.

**Table 3:** Experimental Isotropic Chemical Shifts (δ_iso_) and Chemical Shift Tensors^a^ for Diphenyldiselenide (**1**), and the Cocrystals Studied Herein. All shift values are in ppm

compound	Se site	δ_iso_ ^a^	δ_CSA_ ^b^	δ_11_	δ_22_	δ_33_	η^c^
**1**	1	424.5(0.2)	–239(5.8)	577(6)	511(4)	185.2(4)	0.28(0.03)
**1**	2	349.6(0.2)	–350(9.9)	526(10)	523(8)	–0.4(7)	0.01(0.04)
**1a**	1	548.1(0.2)	281(2)	829(2)	521(1)	294(1)	0.81(0.01)
**1a**	2	521.9(0.3)	–151(4)	660(2)	534(1)	371(1)	0.84(0.01)
**1b**	1	412.2(0.1)	–275.3(2)	566(2)	534(2)	137(1)	0.12(0.01)
**1b**	2	402.9(0.1)	–289.1(2)	550(2)	545(2)	114(1)	0.02(0.01)
**2b**		414.1(0.1)	–312(5)	720(3)	420(3)	102(2)	0.96(0.01)

a δ_11_ ≥ δ_22_ ≥ δ_33_; δ_iso_ = (δ_11_ + δ_22_ + δ_33_)/3.

b Chemical shift anisotropy.

c Asymmetry parameter

In the spectra of compounds **1**, **1a**, and **1b**, two distinct selenium sites are observed, indicating the presence of two crystallographically unique selenium environments. This differentiation is further supported by DFT calculations, which assist in assigning the two selenium sites in these compounds (vide infra).

In the parent compound, diphenyldiselenide (**1**), two distinct crystallographic selenium sites are observed due to intermolecular ChB interactions. One selenium atom (Site 1) acts as a σ-hole donor in the ChB interaction, exhibiting a higher chemical shift (424.5 ppm), while the other selenium atom (Site 2) acts as a ChB acceptor (electron density donor) and shows a lower chemical shift (349.6 ppm). Initially, this chemical shift difference highlights the role of subtle structural features and directional electron-density flow induced by ChB interactions in influencing shielding and deshielding effects at the selenium centers.

To further clarify the relative contributions of intrinsic molecular geometry and intermolecular interactions (XB/ChB), additional gauge-including projector-augmented wave (GIPAW)-DFT calculations were performed on both the isolated molecule and its crystalline geometry. Calculated shifts for the isolated molecule (423.3 ppm for Se1 and 343.2 ppm for Se2) and for the crystalline geometry (439.9 ppm for Se1 and 339.5 ppm for Se2) revealed a relatively modest difference of approximately 16.6 ppm for Se1 and −3.7 ppm for Se2. This indicates that the intrinsic molecular geometry—particularly internal torsion angles and electron distribution—primarily dictates the selenium chemical shifts, while the intermolecular ChB interactions present in the crystal packing contribute in a subtle, yet discernible manner. Thus, the distinct chemical shift environments observed experimentally in the solid-state ^77^Se CP/MAS NMR spectrum predominantly reflect intrinsic molecular conformations, with intermolecular interactions serving as secondary contributors.

Upon cocrystallization with iodine (**a**) to form **1a**, these selenium environments are further modulated by the introduction of strong Se···I halogen bonding and accompanying chalcogen bonding. In **1a**, both selenium sites experience significant deshielding, with their chemical shifts increasing to 548.1 ppm (Site 1 or Se1 in Figure 2a) and 521.9 ppm (Site 2 or Se2 in Figure 2a). Here, Se1 donates electron density to iodine atom, reinforcing the XB interaction, while Se2 maintains its role as a ChB donor (σ-hole donor) in the altered crystal environment. This trend deviates from the observations in **1**, where the σ-hole acceptor site (electron density donor) exhibited a lower chemical shift. A possible explanation is that, despite Se1 acting as an electron density donor in the XB interaction, its close proximity to iodine (2.99 Å) and the strong electronic influence of the iodine atom led to a higher chemical shift compared to Se2. The pronounced shift in both selenium sites underscore the significant impact of σ-hole interactions on the local electronic and magnetic environments, effectively enhancing polarization and reducing electron density around the selenium nuclei.

In contrast, cocrystallization with *p*-DITFB to form **1b** introduces weaker interactions that do not involve direct Se···I contacts. Although the selenium environments are still influenced by ChB interactions, the overall effect is less pronounced. The chemical shifts change from 424.5 and 349.6 ppm in **1** to approximately 412.2 (Site 1 or Se1 in Figure 2b) and 402.9 (Site2 or Se2 in Figure 2b) ppm in **1b**. Although both selenium sites are affected, the absence of a strong direct XB interaction leads to comparatively smaller deshielding effects.

In this case, Se1 acts as a σ-hole donor and Se2 as a σ-hole acceptor in ChB interactions within the unit cell with other selenium atoms. This trend aligns with the observations in **1**, where only Se···Se ChB interactions are present, and the electron density acceptor (σ-hole donor) site exhibits a higher chemical shift. Notably, in the absence of interactions with high-electron-density species—such as the iodine-mediated interaction observed in **1a**—this trend holds: the σ-hole donor site, which accepts electron density, consistently exhibits a higher chemical shift compared to the electron density donor Se site.

A notable observation is that the solid-state ^77^Se CP/MAS NMR spectrum of **1** (Figure 3a) exhibits two distinct resonances at δ_iso_ = 424.5 and 349.6 ppm, whereas only a single signal at δ = 463 ppm is observed in solution.^41^ This discrepancy highlights the influence of the solid-state environment, where intermolecular noncovalent interactions (such as XB and ChB) and crystal packing result in distinct selenium sites, each characterized by a unique local electronic environment. To clarify the relative contributions of intrinsic molecular geometry and intermolecular interactions, additional GIPAW-DFT calculations were conducted on both the isolated molecule and its crystalline geometry. The calculated shifts for the isolated molecule (423.3 ppm for Se1 and 343.2 ppm for Se2) and the crystalline geometry (439.9 ppm for Se1 and 339.5 ppm for Se2) showed relatively modest differences of approximately 16.6 ppm and–3.7 ppm, respectively. This indicates that intrinsic molecular geometry—specifically internal torsion angles and electron distribution—predominantly determines the observed chemical shifts, while intermolecular ChB interactions in the crystal structure have a subtle yet detectable influence. Therefore, the significant 75 ppm chemical shift difference between solid-state and solution spectra primarily arises from conformational changes upon crystallization, further underscoring the exceptional analytical power of ^77^Se SSNMR to probe subtle structural and electronic variations in selenium-containing solids.

^13^C SSNMR spectroscopy (Figures S5 and S6) has proven to be an effective indirect probe for investigating noncovalent interactions, with chemical shift changes providing evidence of cocrystallization through interactions such as XB and ChB.^42–44^ This technique allows us to gain insights into the changes in the crystallographic and electronic environment surrounding carbon atoms, particularly those covalently bonded to selenium (C–Se) in the acceptor moieties. Although the ^13^C nucleus is indirectly involved in each XB or ChB (one bond removed), it is still sensitive to subtle alterations in the electronic and crystallographic environment induced by cocrystallization. These interactions result in observable shifts in the ^13^C NMR spectra, as demonstrated in Figure S5 for compound **1** and cocrystals (Figures S4–S6). Note that compound **2** was not included, as it is a liquid. The ^13^C SSNMR spectra show distinct chemical shifts for the starting materials compared to the cocrystals, reflecting differences in the local electronic environment and confirming the formation of new noncovalent interactions.

### Computational Studies

#### ESP MAP

To understand the σ-hole interactions, electrostatic potential (ESP) maps^45,46^ are frequently used in conjunction with ^77^Se NMR spectroscopy (Figure 4).^47^ The ESP analysis allows one to visualize charge distribution within a molecule, highlighting potential electrophilic or nucleophilic sites. By mapping the electrostatic potential around a molecule, it is possible to pinpoint areas likely to engage in ChBs, offering a visual representation of interaction sites. These maps are particularly valuable for interpreting NMR data, as they correlate observed chemical shifts with specific regions of charge density and potential ChB formation. The integration of ^77^Se NMR spectroscopy and ESP maps provide a comprehensive approach to studying ChBs, providing both experimental and theoretical insights into the chemical and electronic structures of selenium-containing compounds.

**Figure 4:**
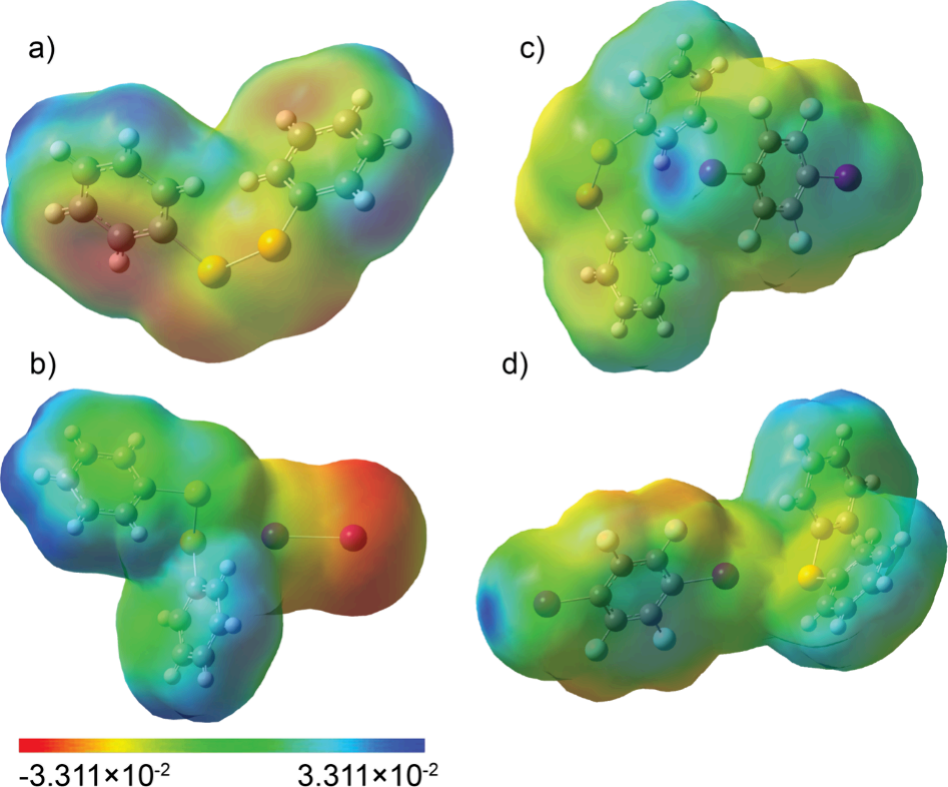
ESP maps of (a) diphenyldiselenide (**1**), (b) its cocrystal with iodine (**1a**), (c) its cocrystal with *p*-DITFB (**1b**), and (d) the cocrystal of diphenylselenide (**2**) with *p*-DITFB (**2b**). The maps illustrate the charge distribution across the molecular surfaces, highlighting regions of electron density (blue) and electron deficiency (red), which correspond to nucleophilic and electrophilic sites, respectively.

In the case of diphenyldiselenide, the chemical shift of selenium appears at 424.5 ppm for the σ-hole donor and 349.6 ppm for the σ-hole acceptor, which is evident from the ESP map of **1**. In the case of cocrystal **1b**, the σ-hole donor appears at 412.2 ppm and the acceptor appears at 349.6 ppm. In the case of cocrystal **1a** the σ-hole acceptor comes at 548.1 ppm, which is more deshielded and is engaged in the halogen bond. In the case of **2b**, the σ-hole acceptor appears at 414.1 ppm, showing a moderated deshielding due to the weaker halogen interaction.

The GIPAW-DFT calculations exhibit a linear correlation between the experimental ^77^Se isotropic chemical shifts (δ_iso_) and the calculated isotropic shielding constants (σ_iso_), as illustrated in Figure 5. For the fully optimized structures (Figure 5a), the correlation is particularly robust, yielding an *R* ^2^ value of 0.982 and demonstrating excellent agreement between the computed and experimental data. The resulting regression equation, Cal. σ_iso_ = −1.188 Exp. δ_iso_ + 1.692 × 10^3^, underscores the method’s predictive capabilities. The calculated chemical shift components derived from the regression equation based on fully optimized structures are summarized in Table 4.

**Figure 5:**
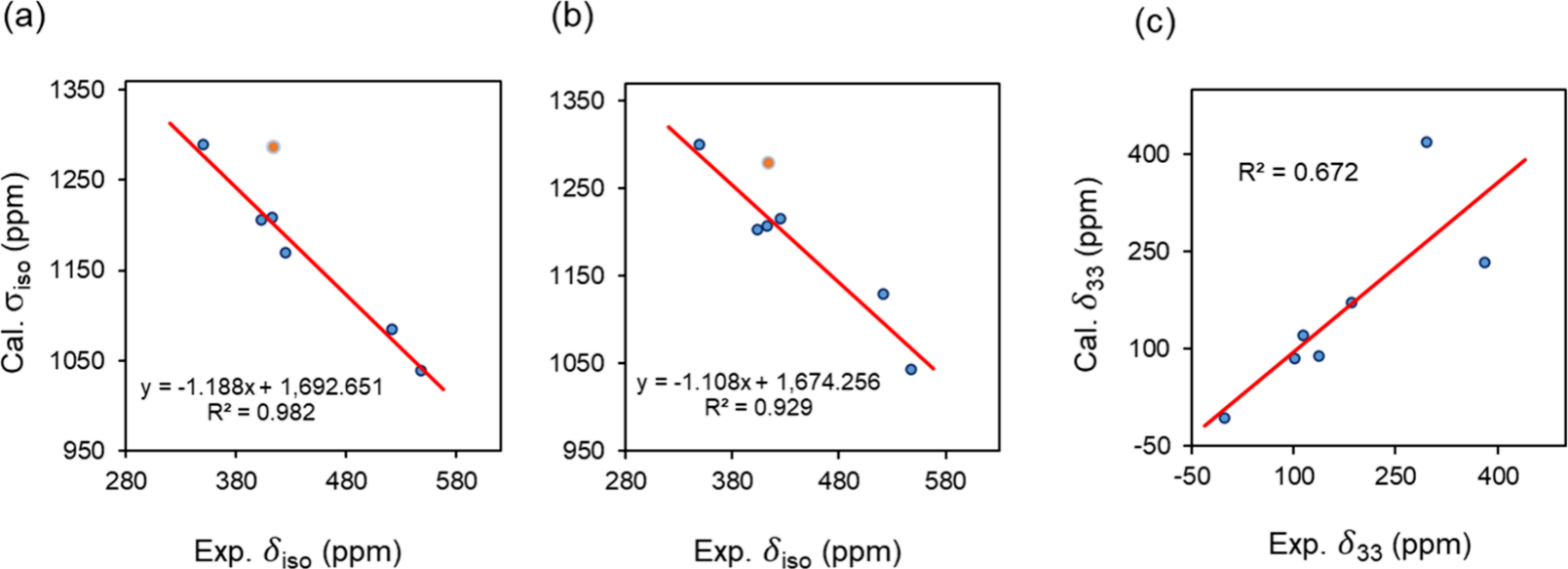
Correlation plots comparing calculated and experimental ^77^Se NMR parameters: (a) isotropic chemical shielding constants (σ_iso_) vs experimental isotropic chemical shifts (δ_iso_) for fully optimized structures. The linear regression excluding the outlier (**2b**, shown as an orange dot) yields Cal. σ_iso_ = −1.188 Exp. δ_iso_ + 1.692 × 10^3^ (*R* ^2^ = 0.982), while including the outlier gives Cal. σ_iso_ = −1.260 Exp. δ_iso_ + 1.737 × 10^3^ (*R* ^2^ = 0.872). (b) σ_iso_ vs δ_iso_ for hydrogen-optimized structures. Excluding the outlier (**2b**), the regression is Cal. σ_iso_ = −1.108 Exp. δ_iso_ + 1.674 × 10^3^ (*R* ^2^ = 0.929); including it: Cal. σ_iso_ = −1.162 Exp. δ_iso_ + 1.707 × 10^3^ (*R* ^2^ = 0.866). (c) Calculated δ_33_ vs experimental δ_33_ for all selenium sites: Cal. δ_33_ = 0.873 Exp. δ_33_ + 7.470 (*R* ^2^ = 0.672).

**Table 4:** Experimental Isotropic Chemical Shifts (δ_iso_) and GIPAW-DFT (Fully Optimized Structure) Calculated ^77^Se SSNMR Parameters for the Compounds Studied Herein^a^. All values are in ppm

compound	Se site	δ_iso,expt_	σ_iso_,_calc_	δ_iso_,_calc_	δ_11_,_calc_	δ_22,calc_	δ_33,calc_	δ_iso,calc_–δ_iso,expt_
**1**	1	424.5	1170.0	439.9	650.3	497.1	172.2	15.3
**1**	2	349.6	1289.3	339.5	622.4	401.7	–5.6	–10.1
**1a**	1	548.1	1038.3	550.7	705.1	528.9	418.2	2.6
**1a**	2	521.9	1084.6	511.7	859.6	442.8	232.8	–10.2
**1b**	1	412.2	1208.3	407.6	669.6	464.5	88.8	–4.6
**1b**	2	402.9	1205.6	409.9	686.4	422.8	120.7	7.0
**2b**	_	414.1	1286.3	357.2	625.6	360.5	85.6	–56.9

a Cal.σ_iso_ = −1.188 Exp. δ_iso_ + 1.692 × 10^3^

The differences between calculated and experimental chemical shifts are generally small in Table 4. For example, **1a**(1) and **1a**(2) exhibit deviations of only 2.6 and −10.2 ppm, respectively. While a larger discrepancy of 56.9 ppm is observed for **2b**, this may reflect the influence of specific intermolecular interactions or structural constraints not fully captured in the model. Further, this difference represents only 1% of the total ^77^Se chemical shift range. Overall, the GIPAW approach employed here is sufficiently accurate for computing ^77^Se NMR parameters in the studied systems. These results are consistent with previous work using GIPAW and (Zeroth Order Regular Approximation) ZORA-DFT methods to accurately predict ^77^Se NMR parameters,^48,49^ confirming the reliability and transferability of such computational strategies.

The GIPAW algorithm,^50^ which reconstructs the all-electron wave function in the presence of a magnetic field, has proven to be an accurate and reliable method for simulating the ^77^Se chemical shift. The principal components of the chemical shift tensor, δ_11_, δ_22_, and δ_33_, are reported following the Maryland convention,^51^ where they are arranged in descending order such that δ_11_ > δ_22_ > δ_33_. The δ_iso_, is calculated as the average of the three principal components:

$$\delta_{\mathrm{iso}}=\frac{\delta_{11}+\delta_{22}+\delta_{33}}{3}$$ i

A strong agreement between the experimental and calculated values across a wide chemical shift range enables the precise assignment of experimental ^77^Se NMR spectra for compounds containing multiple crystallographically distinct Se sites. The calculated ^77^Se NMR parameters effectively differentiate these selenium sites, as demonstrated in compounds, and **1b** (Figure S7), where clear variations in the calculated chemical shifts correlate well with experimental results.

For example, in cocrystal **1a**, two distinct selenium sites exhibit different roles within the XB and ChB interactions. Selenium site 1 (Se1), which participates as a Lewis base in a XB by donating electron density to iodine, exhibits a higher experimental chemical shift of 548.1 ppm, consistent with the calculated GIPAW-DFT value of 550.7 ppm. Conversely, site 2 (Se2), which acts as a Lewis acid in a ChB by interacting with iodine, shows a lower chemical shift in both experimental and calculated values, reflecting its role as an electron density acceptor (vide infra, Figure 6a). A similar trend is observed for compounds **1** and **1b**, where distinct selenium sites are clearly resolved due to their differing bonding roles and electronic environments.

**Figure 6:**
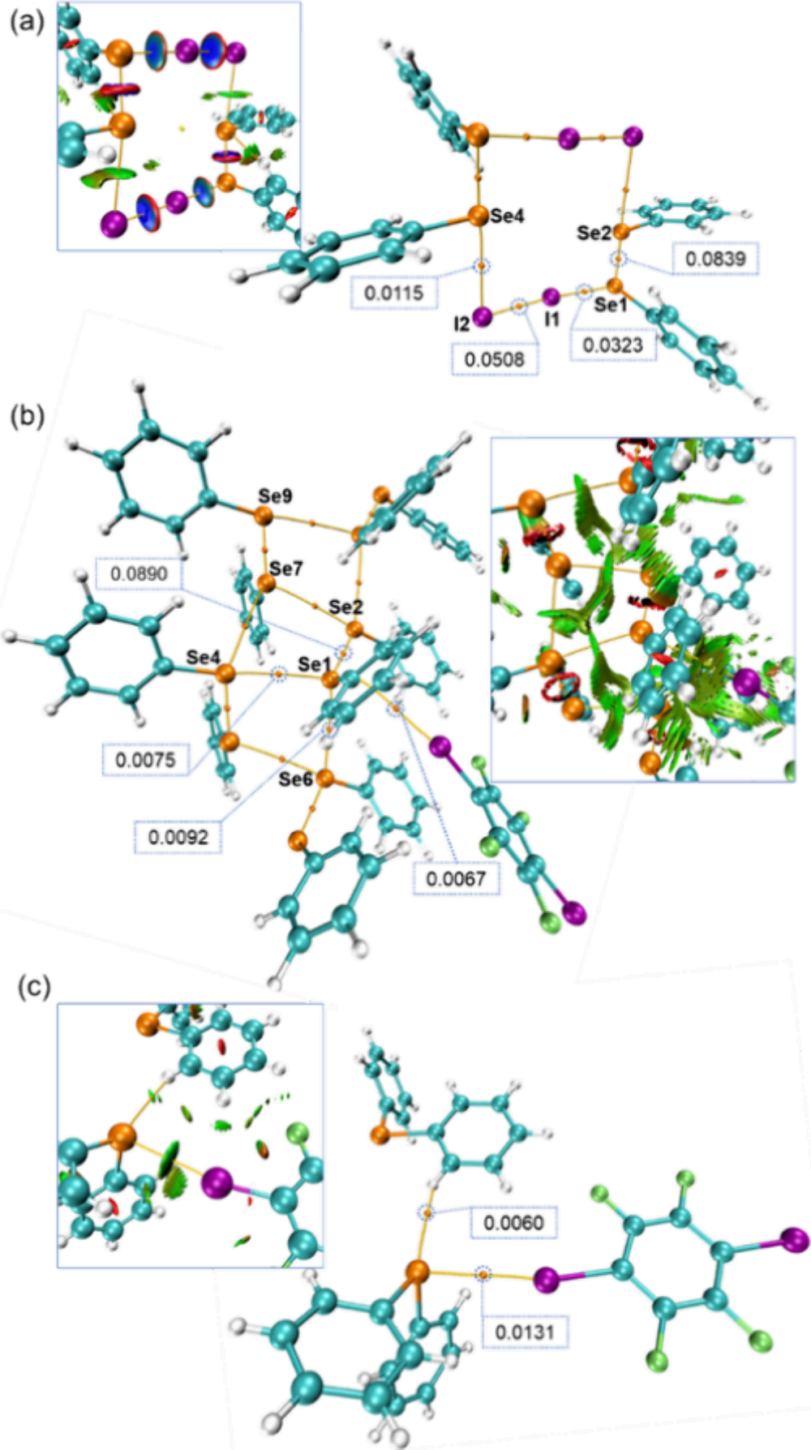
QTAIM and NCI graphs illustrating the selected interactions in the cocrystals of **1a** (a), **1b** (b), and **2b** (c). The NCI graphs are displayed as insets within each QTAIM plot, highlighting the regions of noncovalent interactions. In the NCI plots, green isosurfaces represent weakly attractive interactions (e.g., van der Waals forces, XB, and ChB), red regions indicate repulsive interactions, and blue regions denote strong attractive interactions. The values of electron density at the BCPs are provided in atomic units (a.u.) at selected BCPs, corresponding to data in Table 5.

### QTAIM and NCI Analysis

The nature of intermolecular interactions within the studied systems was investigated using the The Quantum Theory of Atoms in Molecules (QTAIM). QTAIM provides a powerful framework for analyzing noncovalent interactions, such as XB and ChB, as well as covalent bonds, by evaluating key topological parameters at the bond critical point (BCP). Key descriptors include the electron density (ρ_BCP_) and the Laplacian of electron density (∇^2^ρ_BCP_), which indicate interaction strength and bonding type. A positive ∇^2^ρ typically signifies closed-shell interactions (e.g., hydrogen or ionic bonds), while a negative value is associated with shared-shell, covalent interactions. The electron density (ρ_BCP_) and the Laplacian of electron density (∇^2^ρ_BCP_) are particularly informative, as they allow for the characterization of bond strength and bonding type.^52,53^ These data, summarized in Table 5 and illustrated in Figure 6, provide insight into bonding characteristics across the studied systems.

**Table 5:** QTAIM Analysis of the Selected Interactions at the BCPs in the Cocrystals Studied, Highlighting Electron Density (ρ), Laplacian of Electron Density (∇^2^ρ), and Total Energy Density (*H*(*r*)) Values

cocrystal	interaction (BCP)	ρ (a.u.)	∇^2^ρ (a.u.)	*H*(*r*) (a.u.)
**1a**	Se1–Se2 (covalent)	0.0585	0.0478	–0.0121
	I–I (Covalent)	0.0340	0.0514	–0.0025
	I1···Se1 (XB)	0.0336	0.0603	–0.0020
	Se4···I2 (ChB)	0.0115	0.0297	0.0010
**1b**	Se1–Se2 (covalent)	0.0716	0.0282	–0.0204
	I1···π(C) (XB)	0.0075	0.0259	0.0142
	Se1···Se4 (ChB)	0.0089	0.0243	0.0009
	Se1···Se6 (ChB)	0.0123	0.0330	0.0011
**2b**	I···Se (XB)	0.0135	0.0349	0.0011
	Se···H (HB)	0.0060	0.0226	0.0015

To more accurately describe weak chalcogen-bonding interactions, dispersion effects were explicitly included in our QTAIM and NCI analyses using Grimme’s D3-BJ correction scheme. Dispersion interactions are now well-established as essential contributors to chalcogen bonding, particularly in systems involving heavy atoms, as demonstrated in theoretical studies by Bleiholder et. al^54^ and by Huber and co-workers.^55^

In cocrystal **1a**, the bonding environment is multifaceted, involving both covalent and noncovalent interactions. Specifically, Se1 and Se2 are covalently bonded, while Se1 also participates in a XB with iodine, and Se4 forms a ChB with a different iodine moiety. The I–I bond represents the covalent linkage between the iodine atoms within the halogen bond donor. The inclusion of dispersion corrections in the QTAIM analysis slightly altered the electron density parameters, providing a more accurate picture of these interactions.

The Se1–Se2 covalent bond shows the highest electron density at the bond critical point (BCP), with ρ = 0.0585 au, ∇^2^ρ = 0.0478 au, and *H*(*r*) = −0.0121 au, confirming its covalent character. This is consistent with expectations for a typical Se–Se bond, where the negative energy density (*H*(*r*)) reflects strong electron sharing. The I–I covalent bond exhibits an electron density of ρ = 0.0340 au, with a Laplacian of ∇^2^ρ = 0.0514 au, and *H*(*r*) = −0.0025 au The positive Laplacian coupled with a small negative *H*(*r*) suggests a closed-shell covalent interaction, common for halogen–halogen bonds.

The I···Se1 halogen bond has a slightly lower electron density at the BCP (ρ = 0.0336 au) but still shows a comparable Laplacian (∇^2^ρ = 0.0603 au) and *H*(*r*) = −0.0020 au These parameters support the partially covalent nature of the halogen bond, with meaningful charge accumulation and some degree of electron sharing between the iodine and selenium atoms.

Finally, the Se···I2 chalcogen bond is characterized by the weakest interaction, with ρ = 0.0115 au, ∇^2^ρ = 0.0297 au, and a positive energy density (*H*(*r*) = +0.0010 au). The positive *H*(*r*) and lower ρ values confirm that this is a typical noncovalent chalcogen bond, dominated by electrostatic attraction and polarization rather than electron sharing.

In cocrystal **1b**, the I1···π­(C) interaction (ρ = 0.0075 au, ∇^2^ρ = +0.2586 au, *H*(*r*) = +0.0142 au) reflects a typical weak halogen bond, dominated by electrostatic interactions, consistent with the characteristics of halogen−π interactions. Despite the low electron density, the positive Laplacian and Hamiltonian energy density confirm its noncovalent, closed-shell nature.

The Se1–Se2 covalent bond shows a significant electron density (ρ = 0.0716 au, ∇^2^ρ = +0.2819 au, *H*(*r*) = −0.0204 au), demonstrating a shared-shell interaction with weak covalent character. Compared to **1a**, the slightly lower ρ and more positive ∇^2^ρ suggest a marginally more polarized Se–Se bond in **1b**, reflecting a subtle difference in selenium’s bonding environment.

The Se1···Se4 chalcogen bond (ρ = 0.0089 au, ∇^2^ρ = +0.2428 au, *H*(*r*) = +0.0009 au) lies within the weak, closed-shell interaction regime. The presence of a bond path and a BCP confirms the interaction, with the positive Laplacian indicating a net depletion of electron density at the BCP. Se1 again acts as a σ-hole donor, evidenced by angular directionality and its dual role in XB and ChB interactions.

Geometrical and topological data further distinguish the bonding roles of selenium centers: Se1 participates in both halogen and chalcogen bonding as a σ-hole donor, while Se2 primarily serves as an electron donor. The directional Se1···Se4 interaction (C–Se1–Se4 = 87°) and the relative location of the BCP reinforce Se1’s electrophilic nature, while Se2’s interactions remain more nucleophilic. Altogether, these features support selenium’s adaptable bonding behavior across different interaction types in **1b**.

From the QTAIM data (Figure 6c and Table 5), the I···Se interaction in the **2b** cocrystal exhibits features consistent with a halogen bond. The electron density at the bond critical point (ρ_BCP_ = 0.0135 au) is above the typical threshold for noncovalent interactions, indicating a significant interaction between the iodine and selenium atoms. The positive Laplacian (∇^2^ρ_BCP_ = +0.0349 au) reflects electron density depletion in the bonding region, which is characteristic of closed-shell interactions such as halogen bonds. Importantly, the total energy density (*H*(*r*) = +0.0011 au) is slightly positive, suggesting that although the interaction is stabilizing, it lacks significant covalent character and remains within the regime of noncovalent, electrostatic-dominated bonding.

The Se···H interaction also presents a detectable electron density (ρ_BCP_ = 0.0060 au) and a positive Laplacian (∇^2^ρ_BCP_ = +0.0226 au), consistent with weak hydrogen bonding. Its *H*(*r*) **=** +0.0015 au, again slightly positive, indicates a weakly stabilizing, closed-shell interaction, likely contributing modestly to the crystal packing.

Taken together, these QTAIM parameters confirm that the I···Se interaction is the more prominent noncovalent force in cocrystal **2b**, while the Se···H interaction provides secondary stabilization. The positive total energy densities in both cases indicate noncovalent but energetically favorable interactions, dominated by electrostatics rather than covalency.

The Non-Covalent Interaction (NCI) analysis^56,57^ provides complementary insights into the nature of the interactions within the cocrystals beyond what is revealed by QTAIM. As shown in the insets of Figure 6, NCI plots visualize weak noncovalent interactions, such as van der Waals forces, XB, and ChB, by highlighting regions of low electron density and low reduced density gradient (RDG). The green isosurfaces in the NCI plots represent weakly attractive interactions, while the red and blue regions indicate repulsive and strong attractive interactions, respectively. For example, in **1a**, the NCI analysis captures the weak dispersive interactions between neighboring phenyl rings, which play a key role in stabilizing the crystal packing. Similarly, in **1b**, the NCI plots reveal the dispersion interactions in the Se···π and Se···Se contacts, aligning with the QTAIM-detected bond critical points. In **2b**, the isosurfaces emphasize the strong XB interactions between iodine and selenium atoms, further corroborating the QTAIM findings. These visualizations illustrate the spatial extent and distribution of noncovalent interactions, offering a qualitative perspective that complements the quantitative parameters obtained from QTAIM analysis.

#### IQA Analysis

To complement the QTAIM and NCI findings, the Interacting Quantum Atoms (IQA)^58,59^ approach was employed to decompose the total interaction energy between atomic basins into classical electrostatic and quantum mechanical exchange-correlation components. This method provides a quantitative measure of the stabilizing (or destabilizing) contributions to bonding, particularly valuable for noncovalent interactions where electron density-based descriptors may be ambiguous. The results are summarized in Table S1 of the Supporting Information, and are discussed below.

The IQA analysis of cocrystal **1a** provides further insight into the nature of bonding interactions involving selenium and iodine, complementing the QTAIM and NCI findings. For the Se1–Se2 bond in diphenyldiselenide, the IQA-derived total interaction energy (−129.37 kcal/mol) confirms a stabilizing interaction, with the covalent contribution (−144.94 kcal/mol) overwhelmingly dominating (112%), and a small destabilizing noncovalent component (+15.56 kcal/mol), consistent with the QTAIM description of weak covalent character and significant electrostatic influence due to diffuse selenium orbitals. The I1···Se1 halogen bond, identified as moderate by QTAIM, also shows a predominantly covalent character (98.4%) with an interaction energy of–52.87 kcal/mol, reinforcing its partially covalent and stabilizing nature despite low electron density at the bond critical point. In contrast, the Se4···I2 interaction, suspected to be a chalcogen bond, exhibits a much weaker interaction energy (−13.56 kcal/mol) with a nearly pure covalent contribution (100.5%), yet the overall weak and slightly destabilizing nature observed in QTAIM (positive *H*(*r*)) suggests that the IQA “covalency” arises from minimal orbital interactions rather than strong electron sharing. Taken together, these IQA metrics align with the QTAIM interpretation: both Se–Se and I–I are weak covalent bonds with substantial electrostatic contributions, while the I···Se and Se···I contacts show gradations in bond strength and covalency, reflecting their classification as halogen and chalcogen bonds respectively, and illustrating the nuanced bonding behaviors typical of heavy element systems.

The IQA results for the **1b** cocrystal offer quantitative support for the nature and relative strengths of the noncovalent and covalent interactions identified via QTAIM and NCI analysis. The Se1–Se2 interaction, with a total interaction energy of −138.65 kcal/mol and a dominant covalent contribution (112.1%), aligns with the QTAIM indication of a weak covalent bond, reinforcing the conclusion that this is a genuine covalent Se–Se bond with some delocalization due to selenium’s diffuse orbitals. In contrast, the Se1···Se4 chalcogen bond, with an interaction energy of only −5.78 kcal/mol and a similarly dominant covalent contribution (110.7%), is much weaker but still shows signs of orbital overlap, supporting its classification as a directional, σ-hole-driven interaction. Finally, the I···π halogen bond between iodine and the aromatic carbon C9 shows a modest interaction energy of −11.24 kcal/mol, with a larger electrostatic (noncovalent) component (63.7%) compared to the covalent part (36.3%). This energetic profile is consistent with the QTAIM and NCI evidence pointing to a closed-shell, electrostatically dominated halogen bond. Overall, the IQA data corroborate the dual role of selenium as both a σ-hole donor and electron donor in the crystal and reinforce the complementary nature of electrostatics and covalency in stabilizing the halogen and chalcogen bonding motifs in **1b**.

For **2b** cocrystal, IQA analysis quantifies the Se···I interaction energy at–13.90 kcal/mol, with a notably high covalent contribution (−16.90 kcal/mol, 121.6%), partially offset by a small destabilizing noncovalent component (+3.00 kcal/mol, −21.6%), further underscoring the strongly polarized, partially covalent nature of this halogen bond. A secondary interaction between selenium and a neighboring hydrogen atom (Se···H = 3.05 Å) was also detected. Though weaker (−5.32 kcal/mol), this contact is still significant and shows a covalent contribution of 62.6%, suggesting a stabilizing influence through a directional Se···H hydrogen-type contact. Collectively, these results confirm the Se···I halogen bond as the primary interaction driving the cocrystal formation in **2b**, with additional stabilization provided by weaker intermolecular contacts.

## Conclusions

This study offers a detailed and multifaceted understanding of noncovalent interactions in diphenyldiselenide and diphenylselenide cocrystals formed with iodine-based halogen bond donors (I_2_ and *p*-DITFB). Using a combination of experimental SSNMR, X-ray diffraction, and advanced quantum chemical analyses—including GIPAW-DFT, QTAIM, NCI, ETS-NOCV, and IQA energy decomposition—we examined in detail the interplay of halogen and chalcogen bonding in the **1**, **1a**, **1b** and **2b** systems.

The ^77^Se SSNMR chemical shifts provide significant evidence of selenium’s amphiphilic character, revealing a tunable balance between its σ-hole donor and acceptor roles. In diphenyldiselenide (**1**), the σ-hole donor (ChB, δ_iso_ = 424.5 ppm) is significantly more deshielded than the σ-hole acceptor (ChB, δ_iso_ = 349.6 ppm), a 74.9 ppm difference. This trend persists in cocrystal **1b** (with *p*-DITFB), where the σ-hole donor (δ_iso_ = 412.2 ppm) remains more deshielded than the acceptor (δ_iso_ = 402.9 ppm) by 9.3 ppm. In contrast, cocrystal **1a** (with I_2_) exhibits an inversion: the σ-hole acceptor site engaged in a halogen bond (XB, δ_iso_ = 548.1 ppm) is more deshielded than the donor site (ChB, δ_iso_ = 521.9 ppm) by 26.2 ppm. In **2b**, the lone σ-hole acceptor site (XB, δ_iso_ = 414.1 ppm) shows moderate deshielding, consistent with a weaker XB interaction. GIPAW-DFT chemical shift calculations incorporating dispersion correction (PBE0-D3­(BJ)) closely track experimental values (*R* ^2^ = 0.901), confirming the accuracy of the electronic structure modeling.

The QTAIM analysis corroborates these spectroscopic trends, revealing both the partially covalent nature of Se–Se bonding (e.g., ρ = 0.0839 au, ∇^2^ρ = +0.0264 au in **1a**) and the moderate strength of Se···I halogen bonds (e.g., ρ = 0.0323 au in **1a**). The NCI plot visualizations further highlight the role of stabilizing dispersive interactions, including Se···I contacts and π···π stacking between phenyl rings.

Notably, the implementation of IQA energy decomposition allowed us to understand the interatomic interaction energies into covalent and noncovalent components, enabling quantitative benchmarking of interaction types. For example, Se···I halogen bonds were shown to possess non-negligible covalent contributions (up to ∼30% covalency), while Se···H–C and π–π interactions were dominated by electrostatics and dispersion. These insights underscore how even ostensibly weak interactions contribute significantly to crystal stability and supramolecular organization.

Together, these findings demonstrate that subtle variations in XB or ChB partners profoundly influence the selenium chemical environment, which is sensitively captured by ^77^Se SSNMR. The comprehensive integration of experimental and computational approaches—augmented by dispersion-corrected DFT and quantitative IQA analysis—provides a robust framework for understanding and rationally tuning selenium-based noncovalent interactions. This work lays the groundwork for the deliberate design of supramolecular materials leveraging selenium’s dual σ-hole behavior in diverse applications, from molecular electronics to functional crystalline materials.

## Experimental Section

### Materials

Diphenyldiselenide, **1** and diphenylselenide, **2** were purchased from Alfa Aesar. 1,4-diiodotetrafluorobenzene (*p*-DITFB), **b**, and iodine, **a**, were obtained from Sigma-Aldrich. All compounds were used as received without further purification. Reagent-grade solvents and doubly distilled water were used.

### Sample Preparation

Powder crystalline samples of compounds **1a**, **1b**, and **2b** were prepared mechanochemically using a benchtop ball mill (Retsch MM 400). Equimolar amounts of starting materials were used for the cocrystallization process. For the liquid-assisted grinding (LAG) method, small amounts of liquid were added: 0.05 μL/mg of CH_2_Cl_2_ for **1a** and CHCl_3_ for **1b**, while **2b** was prepared without any liquid additive. The ball milling process was conducted at room temperature with a milling frequency of 30 Hz for 40 min, utilizing a 10 mL grinding jar and two stainless steel balls (5 mm outer diameter).

A single crystal of **2b** suitable for SCXRD analysis was obtained by slowly adding a solution of **b** (0.4 mmol) in 2 mL of chloroform to a solution of **2** (0.4 mmol) in chloroform, dropwise. The solvent was allowed to evaporate gradually at room temperature, resulting in the formation of bright white to colorless crystals.

### Powder X-ray Diffraction

PXRD data were acquired using a Bruker D8 Endeavor instrument equipped with a 1 kW Cu Kα radiation source (λ = 1.54056 Å) and a LynxEye XE-T high-speed detector. The measurements were performed at room temperature. Samples were placed securely in a well plate and rotated at a speed of 15 rpm during data collection to ensure even exposure.

### Single-Crystal X-ray Diffraction

A single crystal of compound **2b** was mounted on a thin glass fiber using Parabar oil. SCXRD analysis was carried out on a Bruker AXS SMART diffractometer, which used a Mo Kα radiation source (λ = 0.71073 Å) from a sealed tube. The measurements were conducted at a temperature of 213 K. Raw data were collected and processed using the Bruker ApexIII software.^60^ Unit cell parameters were determined from 36 frames obtained via specific ω scans, with semiempirical absorption corrections applied based on equivalent reflections. The systematic absences and unit cell parameters confirmed the assigned space group. Initial structural solutions were obtained using ShelxT direct methods,^61^ and the structures were refined through full-matrix least-squares techniques using *F* ^2^, aided by ShelXL and ShelXle software.^62^ Hydrogen atoms were positioned geometrically and refined using a riding model.

### Solid-State NMR Spectroscopy

#### Experiments at 60 kHz MAS Rate

Solid-state NMR spectra were measured with a Bruker Avance HD 600 WB NMR spectrometer at *B*_0_ = 14.1 T (ν_0_(^1^H) = 600 MHz) with a triple resonance 1.3 mm MAS NMR probe. Samples were gently ground into fine powders and packed in 1.3 mm o.d. zirconium oxide rotors prior to data collection. The ^1^H and ^13^C chemical shifts are quoted relative to neat tetramethylsilane (TMS) using adamantane as a secondary chemical shift standard. Single-pulse ^1^H NMR spectra were recorded using 90° rf pulses operating at the ^1^H nutation frequency ν_H_ ≈ 185 kHz, 16 accumulated NMR-signal transients, and relaxation delays of 2.0 s. The ^13^C NMR spectra were recorded using ^1^H → ^13^C CP at the double quantum Hartmann–Hahn condition,^63^ ν_H_ + ν_C_ = ν_r_, which involved ramped CP of ν_H_ = 20 ± 5 kHz for ^1^H (ν_C_ = 40 kHz), a 1.5 μs 90° ^1^H pulse, and spinal-64 ^1^H decoupling^64^ at ν_H_ = 150 kHz.

#### Experiments at 10 kHz MAS Rate

A Bruker Avance III spectrometer operating at *B*_0_ = 9.4 T [ν­(^1^H) = 400 MHz] with a triple resonance 4 mm MAS NMR probe was used for the ^77^Se NMR experiments. ^77^Se SSNMR chemical shifts were referenced to solid (NH_4_)_2_SeO_4_, (δ_iso_ = 1040.2 ppm). Samples were gently ground into fine powders and packed in 4 mm o.d. zirconium oxide rotors prior to data collection. The spectra were generally obtained at room temperature. A standard ^1^H → ^77^Se CP pulse sequence with proton decoupling was employed. The π/2 pulse length was optimized to be 3.70 μs, and the CP contact time used was 5000 μs. The recycle delay varied from 10 to 60 s. All solid-state NMR spectra were processed using the TopSpin (version 4.3.0) software package developed by Bruker BioSpin GmbH. Chemical shift tensor components were obtained from ^77^Se SSNMR spectra simulated via the Sola module in Topspin (version 4.3.0).

The ^77^Se chemical shift parameters, including the isotropic chemical shift (δ_iso_), the chemical shift anisotropy parameters based on the Maryland convention, and the principal components of the chemical shift tensor (δ_11_, δ_22_, and δ_33_), were obtained by fitting the spinning sideband patterns using the Solid Lineshape Analysis (SOLA) module within TopSpin and the HBA 1.8.1 software.^40^

### Computational Chemistry

Molecular electrostatic potential (ESP) maps were computed using the B3LYP/LANL2DZ level of theory with Gaussian 03, Revision C.02.^65^ The Cartesian coordinates of the individual ChB donor molecules were derived from their crystallographic information files and used as input data. Electrostatic surface potentials were plotted with isovalues set at MO = 0.02 au and density = 0.0004 au, allowing for the determination of maxima and minima at specific points on the molecule.

DFT calculations were performed to determine the ^77^Se magnetic shielding tensors using periodic boundary conditions as implemented in the CASTEP package (version 20.11).^66–68^ Input files were generated using Materials Studio 5.0. Geometry optimizations and NMR calculations employed the PBE exchange-correlation (XC) functional within the generalized gradient approximation (GGA) framework, augmented with the Grimme D3­(BJ) dispersion correction to account for long-range dispersion interactions.^54,55,69–71^ On the fly pseudopotentials were used in all simulations. Two types of geometry optimizations were performed: (i) full atomic relaxation and (ii) hydrogen-only relaxation, both carried out while keeping the unit cell parameters fixed to the experimental values. The resulting geometries were then used to compute and compare NMR parameters. All CASTEP-based DFT calculations were performed using the ZORA scalar relativistic approach to account for relativistic effects.

Plane-wave cutoff energies and k-point meshes were chosen based on system-specific convergence tests. A cutoff of 600 eV was used for geometry optimization, and 700 eV for NMR shielding calculations. The following Monkhorst–Pack k-point grids were employed:

- Compound **1**: 13 × 9 × 3 grid (70 total points)
- Cocrystal **1a**: 8 × 9 × 8 grid (288 total points)
- Cocrystal **1b**: 8 × 14 × 3 grid (84 total points)
- Cocrystal **2b**: 7 × 14 × 5 grid (126 total points)

All initial crystal structures were obtained from SCXRD data. Calculated GIPAW shielding values were converted to chemical shifts (δ) by linear regression using the best-fit line between experimental δ­(^77^Se) values and computed σ­(^77^Se) values across the data set. This calibration accounts for systematic deviations and ensures accurate prediction of chemical shifts. The optimized CIF files, along with the corresponding CASTEP input files and selected output data, are provided in the data repository referenced in the data availability section.

The Energy Decomposition Analysis, including ETS-NOCV and IQA analyses, was performed using the Amsterdam Density Functional (ADF) 2019 software package.^72^ All calculations employed the PBE0 hybrid functional with the triple-ζ doubly polarized (TZ2P) basis set and included Grimme’s D3-BJ dispersion correction to accurately account for long-range van der Waals interactions. Scalar relativistic effects were treated using the ZORA (Zero-Order Regular Approximation) method. The ETS-NOCV analysis provided insights into the orbital and electrostatic components of Se···Se, Se···I, Se···H, and π···π interactions, while the IQA analysis, extracted from the “Interatomic Contributions” section of the output file, enabled quantitative decomposition of the interaction energy into electrostatic, exchange–correlation (covalent), and dispersion terms. Orbital and energy visualizations were rendered using (Amsterdam Modeling Suite) AMS-View within the AMS-GUI interface.^73^

QTAIM and NCI-RDG analyses were carried out using the Gaussian 09 software package.^74^ Geometry optimizations were performed without symmetry constraints using the B3LYP functional in conjunction with Grimme’s D3-BJ dispersion correction to account for long-range van der Waals interactions. The LANL2DZ basis set was used for heavy atoms (Se and I). The optimized wave functions were subsequently analyzed using the QTAIM to identify bond critical points (BCPs), electron densities (ρ), and to visualize dispersive and noncovalent interactions, including halogen and chalcogen bonding. The resulting isosurfaces were rendered using VMD software.^75^

## Supplementary Material



## Data Availability

Representative input and output files from CASTEP-DFT and QTAIM calculations, and optimized structure files are available in the Zenodo repository at: 10.5281/zenodo.15620208. All experimental/computational data and procedures are available in the ESI.

## Notes

Additional solid-state NMR spectra; additional visualizations of orbital interactions; and additional X-ray crystallographic details (PDF)
