# Supplementary material for: Understanding Non-Covalent Interactions in Diphenyldiselenide and Diphenylselenide Cocrystals Using a Combined 77Se Magic-Angle Spinning Solid-State NMR and Quantum Chemical Analysis Approach
Source: J Phys Chem C Nanomater Interfaces. 2025 Jul 16;129(29):13299–311. doi: 10.1021/acs.jpcc.5c01979 (PMC12302199; doi:10.1021/acs.jpcc.5c01979)
Supplement: Supplementary file 1 [file jp5c01979_si_002.pdf]

Supporting Information

Understanding Non-Covalent Interactions in Diphenyldiselenide and Diphenylselenide Cocrystals Using a Combined ^{77}Se Magic-Angle Spinning Solid-State NMR and Quantum Chemical Analysis Approach

Alireza Nari[a], Sajesh P. Thomas[b], David L. Bryce[a]*, and Brijith Thomas[c]*

[a] Department of Chemistry and Biomolecular Sciences, Centre for Catalysis Research and Innovation, and Nexus for Quantum Technologies, University of Ottawa, Ottawa, Ontario KIN6N5, Canada

[b] Department of Chemistry, Indian Institute of Technology Delhi, New Delhi 110016, India

[c] Chemistry Program, Science Division and Mubadala Arabian Center for Climate and Environmental Sciences, New York University Abu Dhabi, Abu Dhabi 129188, United Arab Emirates

ORCID
Alireza Nari: 0009-0008-1630-5892
David L. Bryce: 0000-0001-9989-796X
Sajesh P. Thomas: 0000-0003-3552-8625
Brijith Thomas:0000-0003-3590-0992

* Authors to whom correspondence may be addressed

David L. Bryce: dbryce@uottawa.ca
phone 613-562-5800

Brijith Thomas: brijiththomas@nyu.edu
phone: 971-2628-4238

Table of Contents

Figure S1: Visualisation of the NOVC interactions………………………………………..	S4
Figure S2: The 1D $^{1}H \rightarrow ^{77}Se$ CPMAS NMR spectra of **1a** at different spinning speed……	S5
Figure S3: Experimental $^{1}H \rightarrow ^{77}Se$ CP/MAS NMR spectra at 10 kHz……………………	S6
Figure S4: ^{1}H one pulse, ^{13}C CPMAS, and ^{1}H-^{1}H BABA 2D spectrum of **1a**……………	S8
Figure S5: ^{1}H one pulse and ^{13}C CPMAS NMR spectra of (i) Ph_2Se_2 (**1**), and (ii) **1a** collected at a spinning speed of 60 kHz…...……………………………………………….	S8
Figure S6: Comparison of the high spinning frequency (i) ^{1}H single-pulse and (ii) ^{13}C CP/MAS solid-state NMR spectra of compound **1** and its cocrystal, **1a** ………………….	S9
Figure S7: Crystal packing diagrams of **1** (i), and cocrystals **1a** (ii), **1b** (iii), **2b** (iv)……..	S11
Figure S8: TEM image of cocrystal 2b…………………………………………………..	S15
Table S1: Crystallographic parameters of Ph_2Se_2 and cocrystals…………………………..	S10

ETS-NOCV Analysis: Qualitative and Quantitative Insights into Bonding

The ETS-NOCV (Extended Transition State-Natural Orbitals for Chemical Valence)[1] method provides a powerful tool for visualizing and quantifying chemical bonding interactions in molecular systems. This approach combines NOCV, which highlights the redistribution of electron density upon bond formation, with ETS, enabling the decomposition of total interaction energy (ΔE_{total}) into its contributing components. Specifically, ΔE_{orb}, the orbital interaction energy, quantifies the extent of electronic contributions to bonding, including both sigma (σ) donation from the Lewis base (nucleophile) to the Lewis acid (σ-hole), and pi (π) back-donation from the Lewis acid to the Lewis base.

The NOCV analysis visualizes regions of electron density accumulation (positive values) and depletion (negative values), illustrating the flow of electron density upon bond formation. By combining NOCV with ETS, the method provides a comprehensive understanding of bonding interactions, integrating qualitative insights from electron density maps with quantitative energy contributions.[2] The total interaction energy can be expressed as:

$$\Delta E_{\text{total}} = \Delta E_{\text{int}} + \Delta E_{\text{orb}} + \Delta E_{\text{Pauli}} + \Delta E_{\text{prep}} + \Delta E_{\text{elast}}$$

where each term represents a specific contribution to the bonding. This combined approach is particularly useful for exploring non-covalent interactions, such as chalcogen and halogen bonding, offering a detailed characterization of both their energetic and orbital nature.

The NCI analyses and orbital visualizations in Figure S1, combined with these quantitative ΔE values, thus demonstrate how the nature and strength of halogen bonding modulate the electron density distribution, influencing the bonding environment in these cocrystalline systems.

IQA data: Quantitative Insights into Interactions

Interacting Quantum Atoms (IQA) energy decomposition analysis was performed using the ADF software package at the PBE0/TZ2P level of theory. The data shown in Table S1 were extracted from the "Interatomic Contributions" section of the output files. Listed are selected pairwise interactions from compound **1**, cocrystals **1a**, **1b**, and **2b**, including interaction distances (in Å), energy components (in Hartree and kcal/mol), and the corresponding covalent and non-covalent contributions. Percent covalency was estimated by the ratio of the absolute covalent component to the total interaction energy.

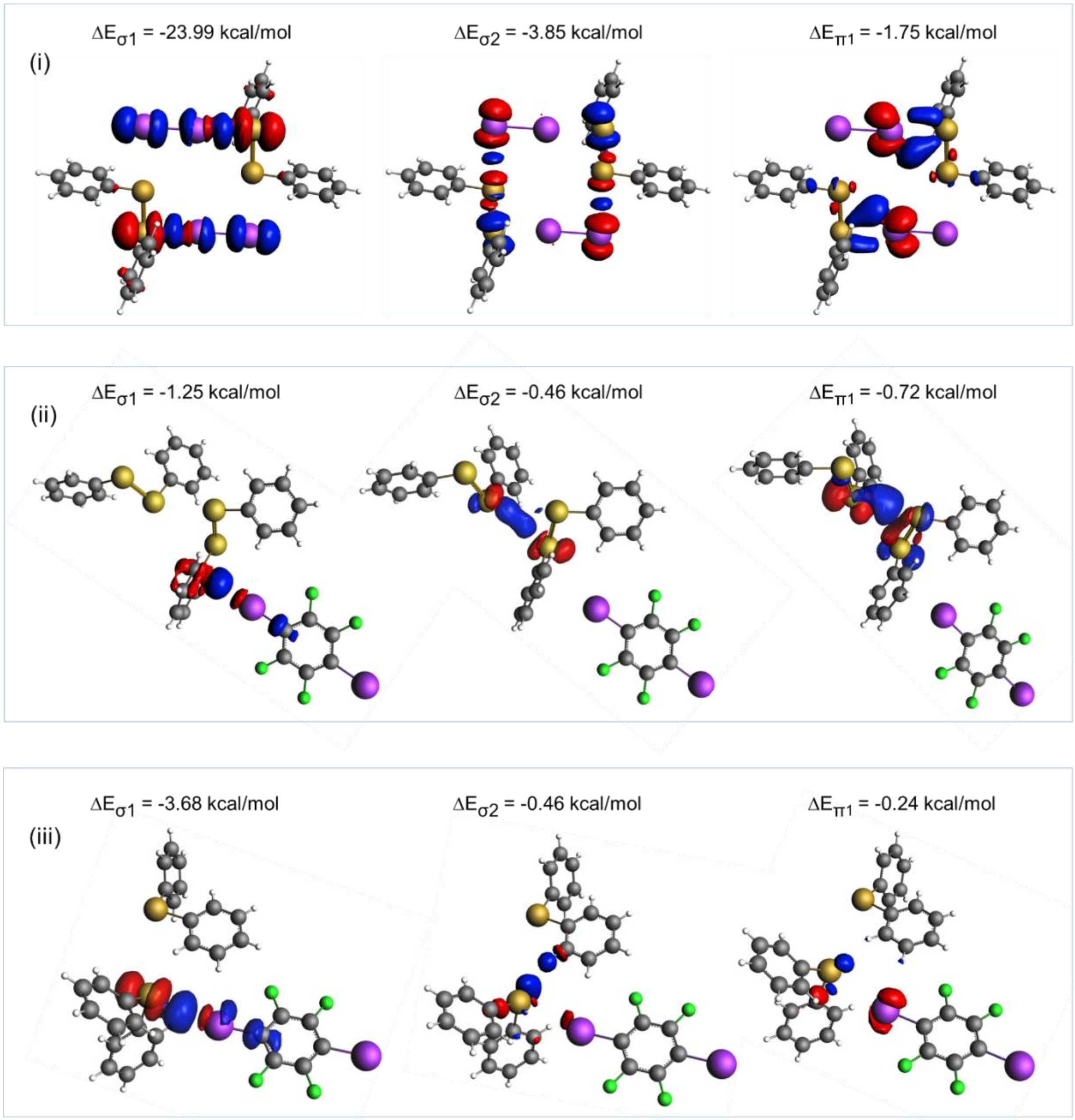

Figure S1. Visualization of the natural orbitals for chemical valence (NOCV) interactions in three cocrystals, analyzed using the ETS-NOCV method to quantify key orbital contributions to intermolecular interactions. Each diagram depicts the most significant NOCV orbital pairs associated with chalcogen and halogen bonding, with corresponding orbital interaction energies (ΔE_{orb}) displayed above each visualization. (i) **1a**, showing electron depletion around the Se–Se bond and accumulation toward the iodine acceptor, supporting the observed bond elongation; (ii) **1b**, illustrating weaker electron redistribution due to π-system interaction; and (iii) **2b**. The orbital interactions are color-coded, with red and blue lobes representing regions of electron accumulation and depletion, respectively.

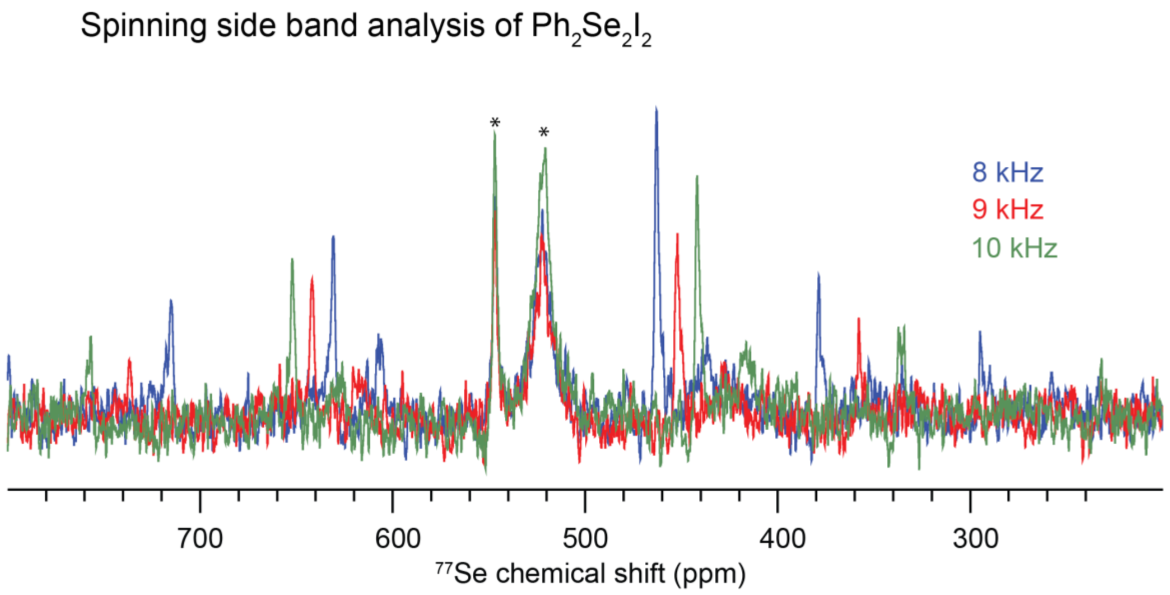

Figure S2. The 1D $^{1}H \rightarrow ^{77}Se$ CPMAS NMR spectra of **1a** at different spinning speeds. The isotropic chemical shifts are observed at 522 ppm and 547 ppm. The peak at 522 ppm is broad, while the peak at 547 ppm is sharp.

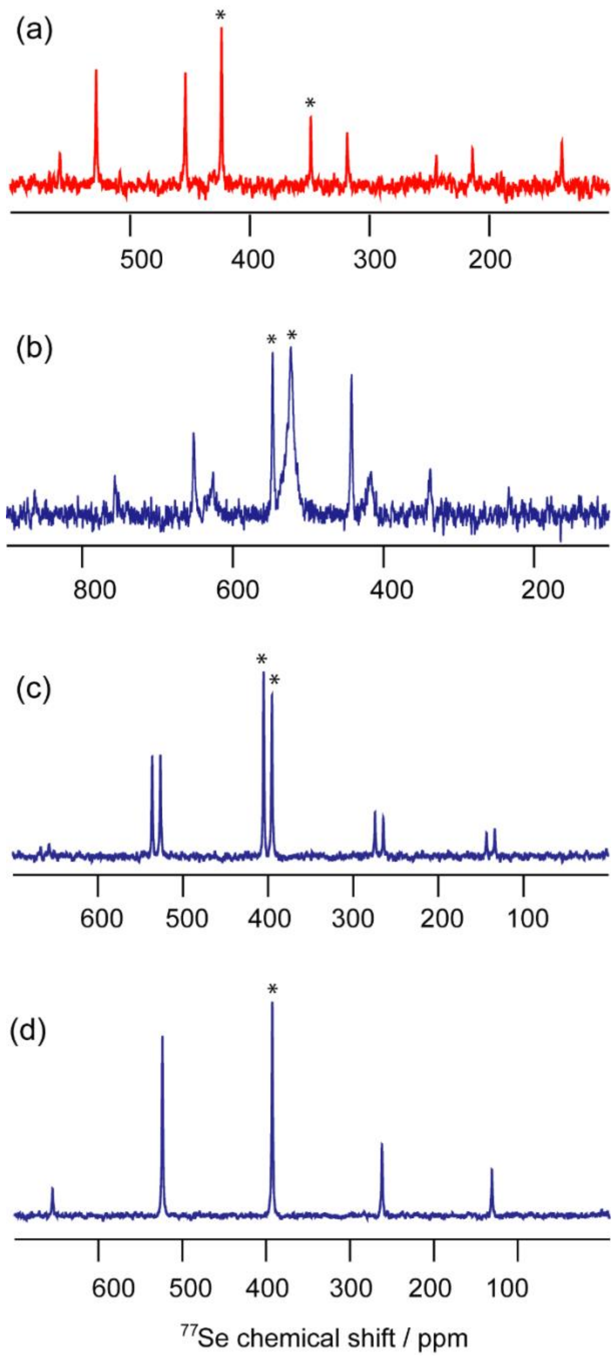

Figure S3. Experimental $^{1}H \rightarrow {}^{77}Se$ CP/MAS NMR spectra of diphenyldiselenide (**1**) and the studied cocrystals, collected at a spinning speed of 10 kHz. Isotropic peaks are indicated with asterisks.

High spinning frequency ^{1}H and ^{13}C SSNMR data

Single pulse ^{1}H SSNMR spectra were recorded using 90° radiofrequency (rf) pulses with a ^{1}H nutation frequency (ν_H) of 185 kHz. Data acquisition involved the accumulation of 16 transients with a relaxation delay (τ_{relax}) of 2.0 seconds.

The ^{13}C spectra were recorded by the ^{1}H $\rightarrow$^{13}C CP at the double quantum Hartmann−Hahn condition,[1] $\nu_H + \nu_C = \nu_r$, which involved ramped CP of $\nu_H = 20 \pm 5$ kHz for ^{1}H ($\nu_C = 40$ kHz), a 1.5 μs 90° ^{1}H pulse, and spinal-64 ^{1}H decoupling[2] at $\nu_H = 150$ kHz.

The double-quantum to single-quantum (DQ–SQ) ^{1}H NMR correlation spectra were recorded using the 2D NMR protocol in the article by Fieke *et.al.*[3] This experiment probes dipolar couplings between ^{1}H nuclei, allowing for the identification of proximities and interactions between protons within the structure. Double-quantum coherence (2QC) excitation and reconversion were achieved using the BABA dipolar recoupling scheme, which spans two rotor periods ($2\tau_r$). This provided 2Q excitation (τ_{exc}) and reconversion (τ_{rec}) intervals of $\tau_{exc}=\tau_{rec}=2\tau_r=33.33$ μs. The BABA scheme ensures efficient recoupling under magic angle spinning conditions, enabling the generation and observation of double-quantum coherences.

The ^{1}H nutation frequency was $\nu_H \approx 185$ kHz, corresponding to 90° dipolar recoupling pulses with a duration of 1.35 μs. This setup ensures precise manipulation of the proton dipolar couplings, crucial for obtaining high-quality 2D NMR spectra.

The 2D NMR acquisition parameters included a τ_{relax} of 2.0 s, with 48 (t_1) × 512 (t_2) time points acquired. Dwell times were set to $\Delta t_1=2\tau_r$ and $\Delta t_2=33.3$ μs, ensuring sufficient resolution in both dimensions. Each t_1 value accumulated 32 transients to enhance the signal-to-noise ratio. The 2D data sets were zero-filled to 256 × 2048 points to improve digital resolution, and apodization

with an exponential Lorentzian broadening of 50 Hz full width at half maximum (FWHM) was applied in both dimensions.

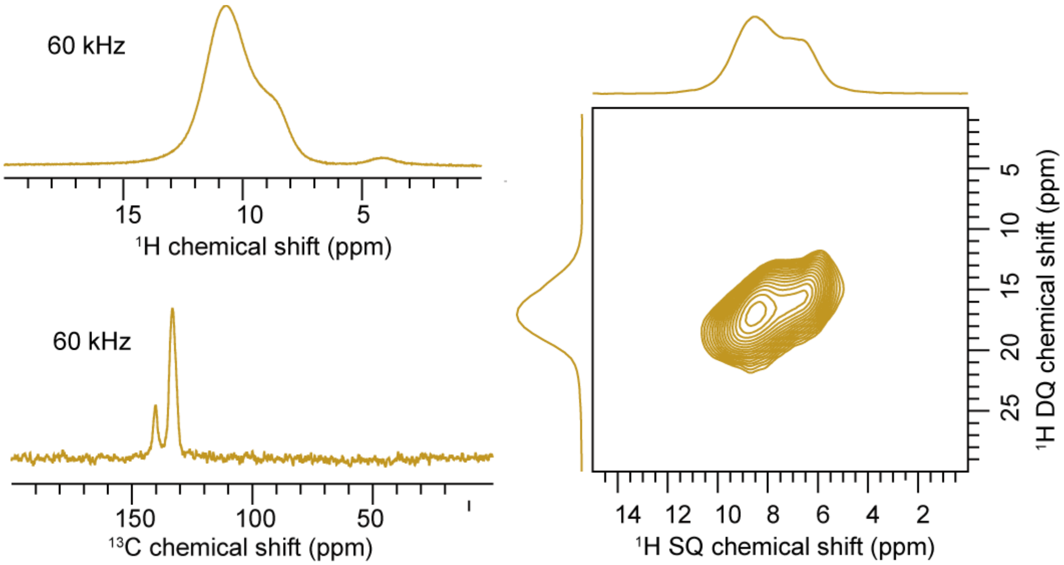

Figure S4. ^{1}H one pulse, ^{13}C CPMAS, and ^{1}H-^{1}H BABA 2D spectrum of **1a**, collected at a spinning speed of 60 kHz. The spectrum reveals correlations between neighboring protons within the aromatic ring.

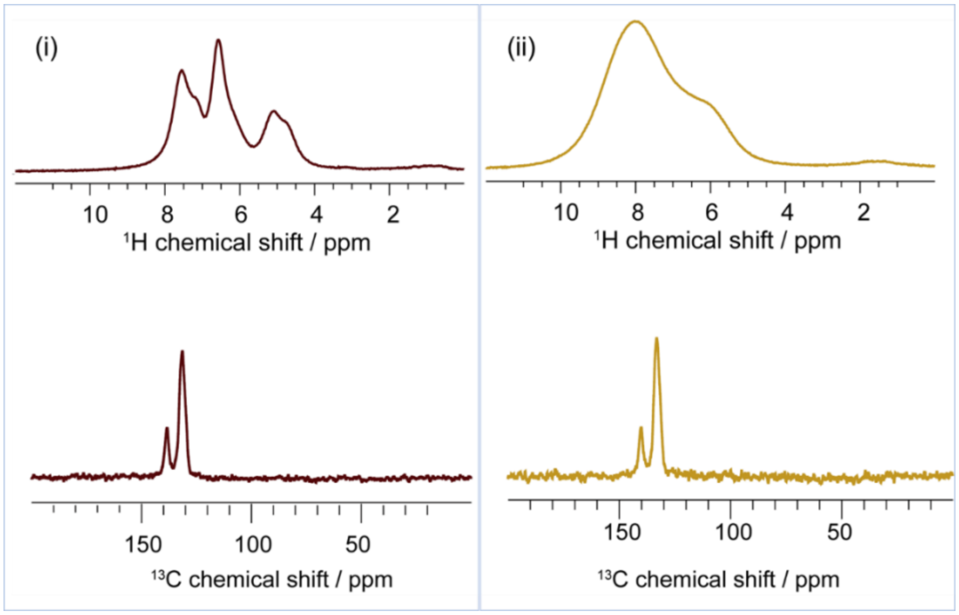

Figure S5. ^{1}H one pulse and ^{13}C CPMAS NMR spectra of (i) Ph_2Se_2 (**1**), and (ii) **1a** collected at a spinning speed of 60 kHz.

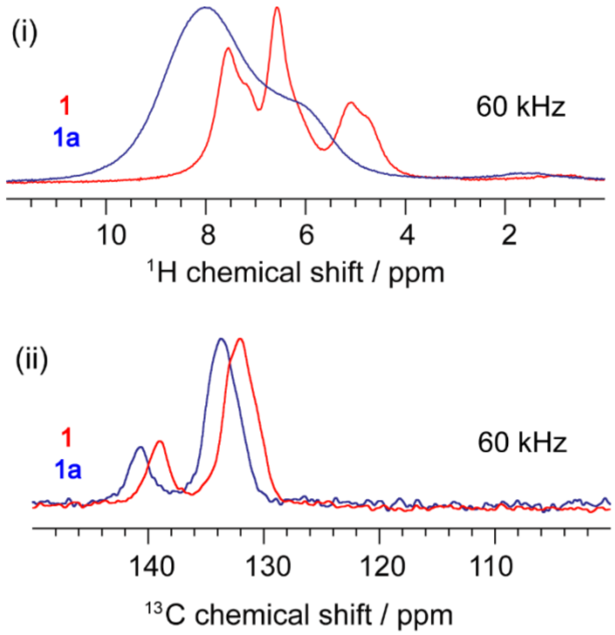

Figure S6. Comparison of the high spinning frequency (i) ^{1}H single-pulse and (ii) ^{13}C CP/MAS solid-state NMR spectra of compound **1** and its cocrystal, **1a**.

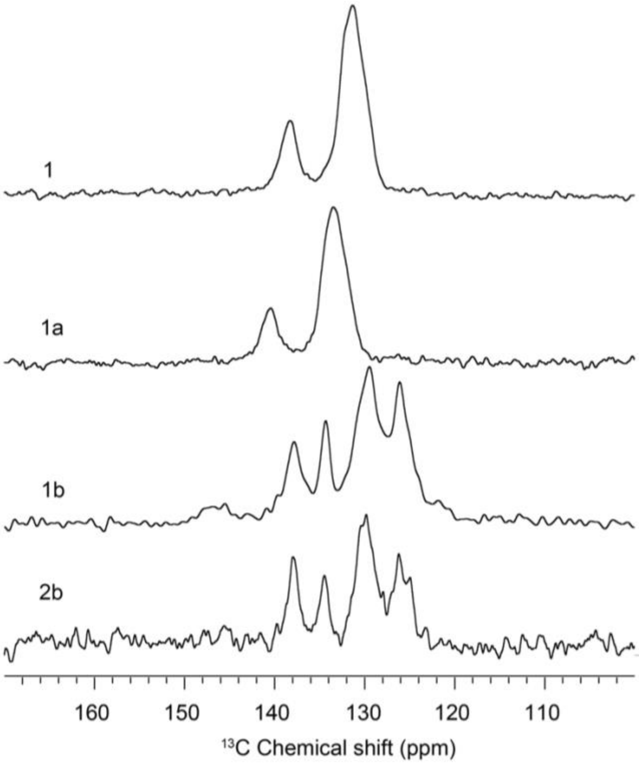

Figure S7. The ^{13}C CP/MAS NMR spectra of diphenyldiselenide (**1**), and cocrystals studied herein, collected at 60 kHz spinning speed. The ^{13}C peaks appearing from the *p*-DITFB moiety are evident in the spectra of **1b** and **2b**.

SCXRD

The crystallographic data for **1a** and **1b** reveal significant structural variations induced by different acceptors (Table S2). Ph_2Se_2 (**1**) crystallizes in the orthorhombic space group $P2_12_12_1$, characterized by a simple and highly symmetrical unit cell with dimensions of a = 5.65 Å, b = 8.29 Å, and c = 24.11 Å. In contrast, its cocrystal with I_2, **1a**, adopts a triclinic structure within the *P*-1 space group, indicative of a less symmetrical arrangement. The unit cell parameters (a = 9.57 Å, b = 9.84 Å, c = 9.98 Å) and the significant deviations of angles from 90° (α = 116.00°, β = 91.18°, γ = 115.57°) indicate a change in packing arrangement associated with the incorporation of iodine, resulting in a more complex molecular environment within the crystal lattice. Similarly, the introduction of *p*-DITFB in **1b** results in a monoclinic structure with the $P2_1/c$ space group, where the unit cell dimensions are a = 10.11 Å, b = 5.67 Å, and c = 26.65 Å, with the β angle slightly deviating from orthogonality at 94.61°, while α and γ remain at 90°. This slight monoclinic distortion indicates that *p*-DITFB influences the molecular packing differently than iodine. These structural changes highlight how the incorporation of different molecules can significantly alter the crystallographic symmetry and unit cell parameters of **1**, leading to variations in physical properties that are critical for potential applications.

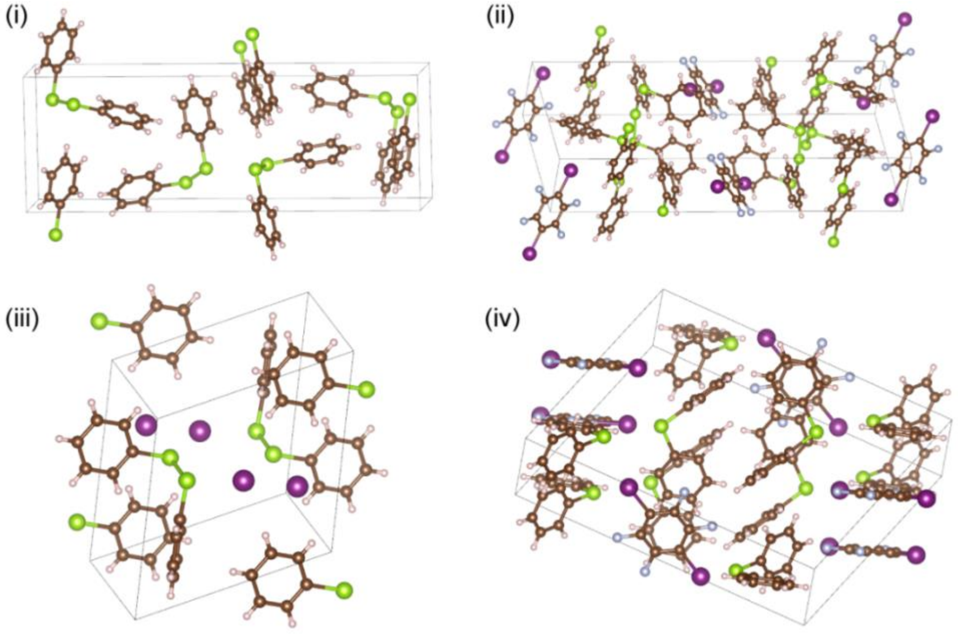

Figure S7. Crystal packing diagrams of **1** (i), and cocrystals **1a** (ii), **1b** (iii), **2b** (iv).

Table S1. Crystallographic parameters of Ph_2Se_2 and cocrystals studied herein.

compound	space group	*a* / Å	*b* / Å	*c* / Å	α / º	β / º	γ / º
**1**	$P2_12_12_1$	5.65	8.29	24.11	90.00	90.00	90.00
**1a**	*P*-1	9.57	9.84	9.98	116.00	91.18	115.57
**1b**	$P2_1/c$	10.11	5.67	26.65	90.00	94.61	90.00
**2b**	$P2_1/n$	13.01	6.15	18.17	90.00	101.80	90.00

Table S2. IQA for Key Se–Se, Se–I, Se–H, and π-Interactions in the Investigated Cocrystals.

**Parameter**	**1**[a]		**1a**			**1b**			**2b**	
	Se1–Se2	Se1–Se25	Se1–Se2	Se4–I2	I1–Se1	Se1–Se2	Se1–Se4	C9–I (π)	Se1–I24	Se1–H50
Distance (Å)	2.307	7.201	2.347	3.588	2.990	2.314	3.752	3.566	3.550	3.053
VeN[b]	-263.380	-160.264	-258.965	-264.305	-317.986	-262.405	-162.657	-49.406	-267.081	-5.868
VNe[c]	-263.014	-160.064	-258.339	-265.805	-317.966	-262.651	-162.576	-46.956	-267.302	-6.614

Vee (Coulomb)[d]	261.295	159.796	256.724	264.350	317.202	260.648	162.209	49.162	265.869	6.585
VNN[e]	265.127	160.532	260.605	265.760	318.748	264.435	163.025	47.188	268.520	5.894
VCoulomb (Total)[f]	0.0277	0.0003	0.0248	0.0001	-0.0013	0.0266	0.0010	-0.0114	0.0048	-0.0032
Vee (Exchange)[g]	-0.2492	-0.0104	-0.2310	-0.0217	-0.0829	-0.2476	-0.0102	-0.0065	-0.0269	-0.0053
Vee (Total)[h]	261.046	159.786	256.493	264.328	317.119	260.401	162.198	49.155	265.842	6.579
Einter (kcal/mol)[i]	-138.99	-6.31	-129.37	-13.56	-52.87	-138.65	-5.78	-11.24	-13.9	-5.32
Covalent part (kcal/mol)	-156.35	-6.51	-144.94	-13.63	-52.03	-155.36	-6.41	-4.08	-16.9	-3.33
Covalent part (%)	112.50%	103.10%	112.00%	100.50%	98.40%	112.10%	110.70%	36.30%	121.60%	62.60%
Non-Covalent part (kcal/mol)	17.36	0.19	15.56	0.07	-0.84	16.71	0.63	-7.17	3.00	-1.99
Non-Covalent part (%)	-12.50%	-3.10%	-12.00%	-0.50%	1.60%	-12.10%	-10.70%	63.70%	-21.60%	37.40%

a: The numbering are from the cif file from the crystallographic data. The optimized cife file is deposited align other manuscript data. All other numbering is corresponded to the Figure 7 in the main text.

b: Coulomb interaction between the electrons of A and the nucleus of B in Hartree

c: Coulomb interaction between the nucleus of A and the electrons of B in Hartree

d: Coulomb interaction between the electrons of A and B in Hartree

e: Coulomb interaction between the nuclei of A and B in Hartree

f: VeN+VNe+Vee(Coulomb)+VNN in Hartree

g: exchange interaction between the electrons of A and B in Hartree

h: Vee(Coulomb)+Vee(Exchange) in Hartree

i: total interaction energy between A and B, i.e. VCoulomb(total)+Vee(exchange) in kcal/mol

TEM image

High-resolution transmission electron microscopy (HRTEM) images were acquired using a Thermo Fisher Scientific (TFS) Talos F200X scanning/transmission electron microscope (S/TEM). This instrument offers a lattice-fringe resolution of 0.14 nm and operates at an accelerating voltage of 200 kV. Imaging was performed with a CETA 16M camera. The samples were prepared by placing a drop of diluted particle solution onto a holey carbon film supported by a copper grid, followed by drying overnight at room temperature (298 K).

TEM images of compound **2b** prepared by mechanochemical ball milling. The observed particle sizes range from ~1 × 2 × 1.5 μm to 3 × 5 × 2 μm and 3 × 2 × 4 μm, confirming the formation of micron-sized crystallites as expected from solid-state synthesis. These images provide direct evidence of the bulk morphology of the materials following the ball-milling process.

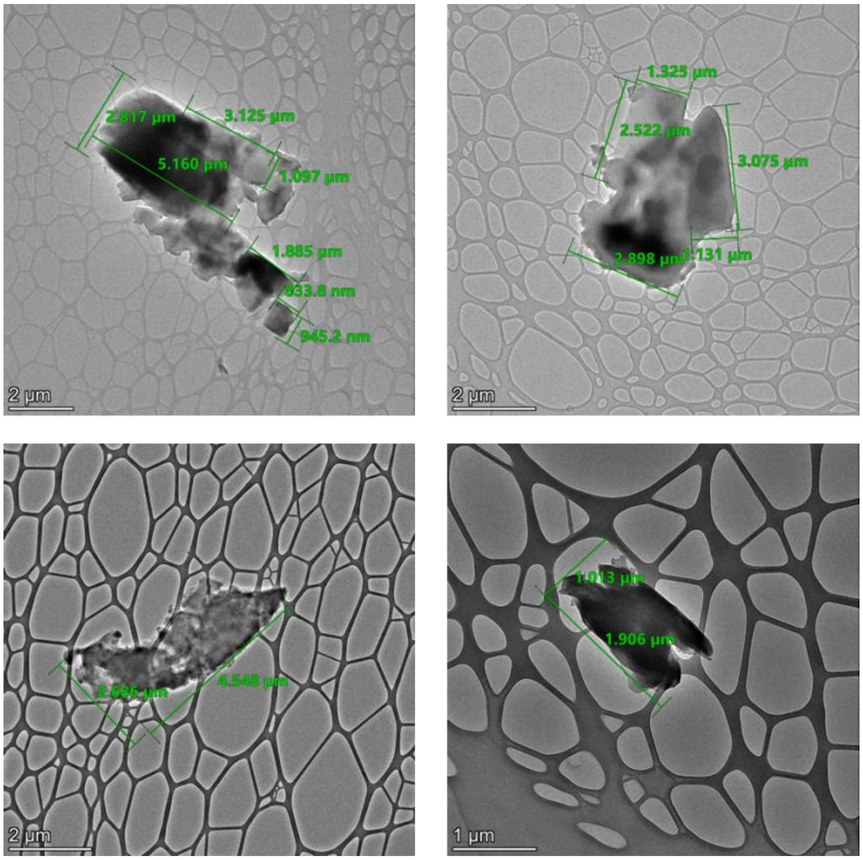

Figure S8. The TEM images of the 2b cocrystal, captured from various orientations showing the crystal size.

Reference

(1) Mitoraj, M.; Michalak, A. Natural Orbitals for Chemical Valence as Descriptors of Chemical Bonding in Transition Metal Complexes. *Journal of Molecular Modeling* **2007**, *13* (2), 347–355.

(2) Mitoraj, M. P.; Michalak, A.; Ziegler, T. A Combined Charge and Energy Decomposition Scheme for Bond Analysis. *J. Chem. Theory Comput.* **2009**, *5* (4), 962–975.

(3) Feike, M.; Demco, D. E.; Graf, R.; Gottwald, J.; Hafner, S.; Spiess, H. W. Broadband Multiple-Quantum NMR Spectroscopy. *Journal of Magnetic Resonance, Series A* **1996**, *122* (2), 214–221.
